# A novel murine model of rhinoscleroma identifies Mikulicz cells, the disease signature, as IL-10 dependent derivatives of inflammatory monocytes

**DOI:** 10.1002/emmm.201202023

**Published:** 2013-04-02

**Authors:** Cindy Fevre, Ana S Almeida, Solenne Taront, Thierry Pedron, Michel Huerre, Marie-Christine Prevost, Aurélie Kieusseian, Ana Cumano, Sylvain Brisse, Philippe J Sansonetti, Régis Tournebize

**Affiliations:** 1 Institut Pasteur, Génotypage des Pathogènes et Santé PubliqueParis Cedex, France; 2 Institut Pasteur, Pathogénie Microbienne MoléculaireParis Cedex, France; 3 INSERM U786Paris, France; 4 Institut Pasteur, Unité de Recherche et d'Expertise Histotechnologie et PathologieParis Cedex, France; 5 Institut Pasteur, Plateforme de Microscopie UltrastructuraleParis Cedex, France; 6 Institut Pasteur, Unité du Développement des LymphocytesParis Cedex, France; 7 INSERM U668Paris, France; 8 Microbiologie et Maladies Infectieuses, Collège de FranceParis Cedex, France

**Keywords:** IL-10, inflammatory monocytes, Klebsiella, Mikulicz cell, rhinoscleroma

## Abstract

Rhinoscleroma is a human specific chronic disease characterized by the formation of granuloma in the airways, caused by the bacterium *Klebsiella pneumoniae* subspecies *rhinoscleromatis*, a species very closely related to *K. pneumoniae* subspecies *pneumoniae*. It is characterized by the appearance of specific foamy macrophages called Mikulicz cells. However, very little is known about the pathophysiological processes underlying rhinoscleroma. Herein, we characterized a murine model recapitulating the formation of Mikulicz cells in lungs and identified them as atypical inflammatory monocytes specifically recruited from the bone marrow upon *K. rhinoscleromatis* infection in a CCR2-independent manner. While *K. pneumoniae* and *K. rhinoscleromatis* infections induced a classical inflammatory reaction, *K. rhinoscleromatis* infection was characterized by a strong production of IL-10 concomitant to the appearance of Mikulicz cells. Strikingly, in the absence of IL-10, very few Mikulicz cells were observed, confirming a crucial role of IL-10 in the establishment of a proper environment leading to the maturation of these atypical monocytes. This is the first characterization of the environment leading to Mikulicz cells maturation and their identification as inflammatory monocytes.

## INTRODUCTION

Rhinoscleroma is a human-specific chronic, progressive granulomatous infection of the upper airways, which may eventually lead to death or severe sequelae (Hart & Rao, 2000). Although considered a rare disease, it is endemic in poor populations of Eastern Europe, Middle East, tropical Africa, South and Central America and South East Asia. Current therapies involve surgery and/or prolonged antibiotic treatment, but a high relapse rate is often observed (Gaafar et al, 2011). The etiologic agent of rhinoscleroma is *Klebsiella pneumoniae* subsp. *rhinoscleromatis* (hereafter named *K. rhinoscleromatis*), a bacterium very closely related to the well known pathogen *K. pneumoniae* subsp. *pneumoniae* (hereafter named *K. pneumoniae*). *K. rhinoscleromatis* has been distinguished from *K. pneumoniae sensu stricto*-based on multilocus sequence typing and biochemical features (Brisse et al, 2009) and on the fact that it causes rhinoscleroma. On the contrary, *K. pneumoniae* is a major cause of hospital-acquired infections such as urinary and respiratory tract infections and bacteremia as well as community-acquired infections such as pneumonia and pyogenic liver abscess (Podschun & Ullmann, 1998; Shon et al, 2013).

The pathogenesis of rhinoscleroma remains poorly understood mainly because of the lack of characterized *in vitro* and *in vivo* models. As a consequence, the literature mainly describes reports of clinical cases. More than 16,000 cases have been reported since 1960, but this number is considered to be an underestimate, as most of them are not clearly identified and/or not reported (van Rentergheim et al, 1993). It is now thought that genetic predisposition contributes to the development of this illness (De Pontual et al, 2008). The disease evolves through three overlapping stages (Hart & Rao, 2000). In the catarrhal stage, bacterial invasion of the subepithelial layer triggers a classical non-specific inflammatory reaction with polymorphonuclear cells recruitment, bacterial phagocytosis, incomplete digestion of bacteria and finally cell death leading to release of bacteria into tissues. The proliferative stage is characterized by the appearance of Mikulicz cells, a hallmark of this disease. These cells are large foamy histiocytes, *i.e.* macrophages with size of up to 100 µm that are unable to digest phagocytozed bacteria, which persist in massively enlarged vacuoles. They appear while *K. rhinoscleromatis* is able to invade and multiply within the subepithelium (Canalis & Zamboni, 2001). The sclerotic stage is characterized by granulomatous masses that result from scarring of chronically infected upper airways.

The histiocytic nature of Mikulicz cells was demonstrated by immunocytochemical staining using markers for alpha-1-antitrypsin and alpha-1-globulin, which excluded their plasmocytic origin (Gaafar et al, 1979; 2000). Histiocytes belong to the monocyte lineage, which is subdivided into two main subsets: the resident monocytes and inflammatory monocytes (Geissmann et al, 2003). They are both found in peripheral blood under steady-state conditions. However, under inflammatory conditions, inflammatory monocytes are recruited to the inflamed tissue where they can differentiate into resident monocytes, macrophages or dendritic cells. Their recruitment depends on a signal mediated by the chemokine receptor 2 (CCR-2) (Kurihara et al, 1997; Kuziel et al, 1997; Lu et al, 1998; Palframan et al, 2001; Serbina & Pamer, 2006; Shi & Pamer, 2011) and their differentiation depends on the cytokine environment. These phenomena have been convincingly demonstrated in infections with *Toxoplasma gondii* (Robben et al, 2005), *Leishmania major* (Sunderkötter et al, 2004), *Entamoeba histolytica* (Helk et al, 2013), *Listeria monocytogenes* (Drevets et al, 2004; Sunderkötter et al, 2004), *Salmonella typhimurium* (Rydström & Wick, 2007), *Streptococcus pneumoniae* (Winter et al, 2009) or viral infections (Zheng & Atherton, 2005; Lin et al, 2008).

Very little is known about the molecular and cellular mechanisms underlying this disease. Mikulicz cells are only documented in rhinoscleroma, suggesting a central role in creating the immunopathological environment that sets the stage for chronic granulomatous inflammation (Canalis & Zamboni, 2001). There are few reports of infection of an animal model by *K. rhinoscleromatis* describing the formation of Mikulicz cells in either mouse (Steffen & Smith, 1961), rat (Gaafar et al, 2000) or rabbit (Talaat et al, 1978). Yet, the precise description of Mikulicz cells is not known, nor how they are recruited and what are the factors required for their maturation. In this work, we successfully developed and characterized a mouse model recapitulating a major step of the disease: the formation of Mikulicz cells. Further, our study identifies for the first time Mikulicz cells as inflammatory monocytes. Using different genetic mouse strains, we characterized their kinetics of recruitment from the bone marrow to the lungs and show that this recruitment is independent from a CCR2-mediated signal. Moreover, our data show that interleukin-10 (IL-10) is highly expressed upon infection with *K. rhinoscleromatis* and that it plays a crucial role in the phenotypic maturation of Mikulicz cells and thereby in rhinoscleroma pathogenesis.

## RESULTS

### A mouse model of rhinoscleroma recapitulates the formation of Mikulicz cells

**Figure 1:**
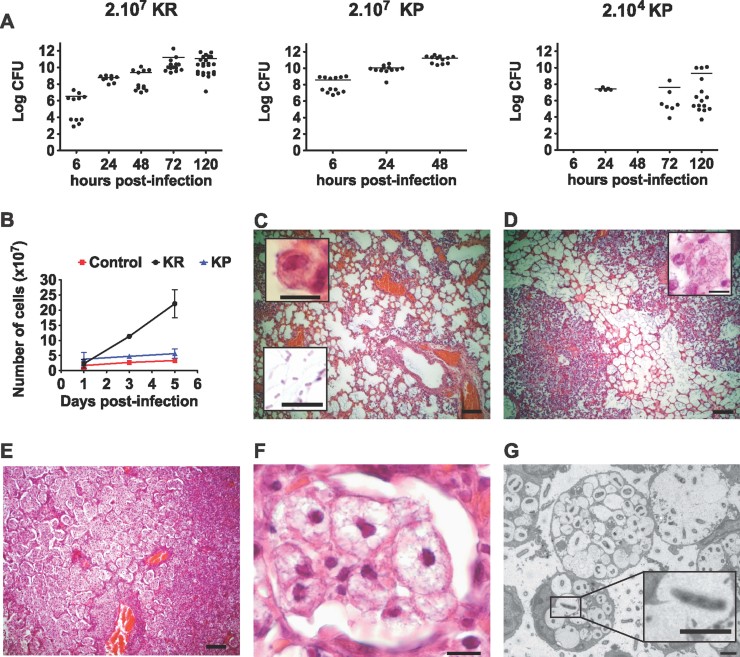
Colonization and histology of BALB/c lungs infected by *K. rhinoscleromatis.* A. Bacterial load in lungs of mice infected with 2.10^7^ *K. rhinoscleromatis* (left), 2.10^7^ Kp52.145 (centre) or 2.10^4^ Kp52.145 (right). Data show log CFU/organ from 5 to 23 mice. Means are indicated as line. B. Quantification of the number of cells present in lungs of saline-injected mice, mice infected with 2.10^7^ *K. rhinoscleromatis* (KR) or 2.10^4^ Kp52.145 (KP). C–F. Lungs of mice infected by 2.10^7^ *K. rhinoscleromatis* were resected 1, 3 and 5 days post-infection and observed by histology. One day post-infection (C) lungs presented a moderate inflammation with intact alveoli containing few alveolar macrophages (inset top) and numerous bacilli (inset bottom). Three days post-infection (D) non-specific inflammatory lesions increased and a few spumous histiocytes appeared (inset). Five days post-infection (E) two types of inflammatory lesions could be seen and distinguished based on their brightness. The right of the picture is composed of darker features that corresponded to classical abscessed lesions with numerous polymorphonuclear cells. The middle and left part of this panel were brighter and corresponded to the reaction implicating foamy histiocytes. A zoom into this region (F) revealed intact alveoli filled exclusively with big spumous histiocytes. Scale bars: C, D and E, 100 µm; insets and F, 10 µm. G. Ultrastructural features of lungs of mice after 4 days of infection with *K. rhinoscleromatis*. Lungs revealed three types of Mikulicz cells, which seem to correspond to three different maturation steps. Mikulicz cells first presented few individual vacuoles (bottom cell) that tended to fuse during maturation (middle and inset) before forming a single enlarged vacuole (top right). Scale bars: 2 µm.

To better understand the pathophysiology of rhinoscleroma we sought to develop a murine model reproducing the human disease, in particular the formation of Mikulicz cells. With the aim of comparing *K. rhinoscleromatis* infection with the well-characterized pulmonary pathophysiology of *K. pneumoniae*, we first evaluated the capacity of the bacteria to colonize the lungs of BALB/c mice following intranasal infection with 2.10^7^ bacteria of either *K. rhinoscleromatis* or *K. pneumoniae* strain Kp52.145. The bacterial load of mice infected with *K. rhinoscleromatis* gradually increased to reach 10^11^ bacteria per organ in 3 days while this amount was reached in 2 days in animals infected with Kp52.145, before succumbing from the infection (Fig 1A). To follow both infections with the same kinetic, we therefore used thereafter a lower inoculum of 2.10^4^ Kp52.145 that caused a slow increase of the bacterial load over 5 days of infection.

Macroscopic observation of the lungs revealed a striking volume increase in the *K. rhinoscleromatis*-infected animals with time (Supporting Information Fig 1). Five days post-infection *K. rhinoscleromatis*-infected lungs were edematous with gray/brownish regions of different sizes ranging from foci of 1 mm in diameter to the whole lobe. In contrast, lungs infected by Kp52.145 showed the same homogenous texture and color as controls, suggesting that this lower Kp52.145 inoculum induced a lower inflammation. To verify whether an increase in cell number could account for the increased lung volume, we determined the total number of lung cells during the course of infection. Five days post-infection with *K. rhinoscleromatis* or Kp52.145, we observed respectively a 10- and 2-fold increase in the total lung cell number (Fig 1B). We concluded that lung volume increase was a specific characteristic of *K. rhinoscleromatis* infection that can be explained in part by a higher cell number, consistent with the edematous process observed macroscopically.

Histological observation 1 day post-infection showed that animals infected by *K. rhinoscleromatis* presented a moderate inflammation characterized by intact alveoli containing numerous bacilli, infiltration of polymorphonuclear cells and macrophages around the vessels (Fig 1C). Three days post-infection, non-specific inflammatory lesions increased and some Mikulicz cells appeared (Fig 1D). Five days post-infection, almost no empty alveoli were visible and two types of inflammatory lesions were observed. First, some regions of the lungs displayed classical abscessed lesions with numerous polymorphonuclear cells (Fig 1E, right side). Second, and more strikingly, many alveoli with an intact epithelial layer were filled almost exclusively with cells presenting features of Mikulicz cells (Fig 1E, left side and Fig 1F). They were large, up to 100 µm, with a pale cytoplasm and an extensive vacuolization. Their nucleus was hyperchromatic, compressed, and often located at the periphery of the cell. Further analysis of these features was performed by electron microscopy (Fig 1G). Mikulicz cells presented a large number of vacuoles, often containing one or several bacilli. Cell size and extent of vacuolization allowed to distinguish three sub-types of Mikulicz cells morphology. First, small Mikulicz cells containing up to 15 vacuoles, with a nucleus generally visible on the side of the cell; second, Mikulicz cells with up to 60 vacuoles; and third, cells containing a unique giant vacuole. Protrusions of a vacuole into a neighboring one were frequently observed. The unique giant vacuole of the third type of Mikulicz cells probably represents a later stage in Mikulicz cells maturation, resulting from the fusion of all vacuoles. The process of vacuole merging was associated with an enlargement of the total cell volume and a reduction of the nucleus. Altogether, these observations showed that this animal model recapitulated an essential feature of rhinoscleroma and is thus a useful tool for its characterization.

### The inflammatory response against *K. rhinoscleromatis* is characterized by inflammatory monocytes recruitment

**Figure 2:**
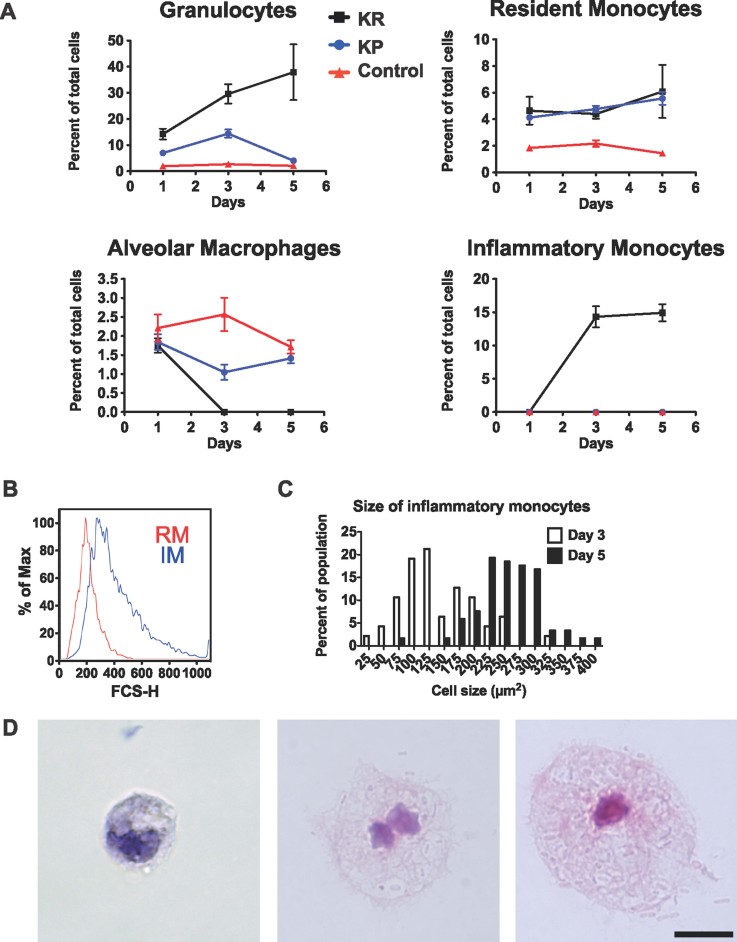
Kinetics of cells recruitment after infection with *K. rhinoscleromatis* and Kp52.145. A. Lung cells of BALB/c mice infected with 2.10^7^ *K. rhinoscleromatis*, 2.10^4^ Kp52.145 or saline-injected controls were isolated 1, 3 and 5 days post inoculation and stained for granulocytes (Gr1^+^ F4/80^−^ CD11b^+^ CD11c^−^), resident monocytes (Gr1^−^ F4/80^+^ CD11b^+^ CD11c^−^), alveolar macrophages (Gr1^-^ F4/80^+^ CD11b^−^ CD11c^+^) or inflammatory monocytes (Gr1^+^ F4/80^+^ CD11b^+^ CD11c^−^). Results show the percentage of each cell population among the total lung cells. Data are mean ± SEM and represent between 6 and 12 mice for each point from at least three independent experiments. B. Forward scatter histogram of resident monocytes (RM, red) and inflammatory monocytes (IM, blue). C. Quantification of sorted inflammatory monocytes size. Cell size was measured 3 (white bars, *n* = 49) and 5 (black bars, *n* = 119) days post-infection. D. Three and five days post-infection by *K. rhinoscleromatis,* cells were isolated and the inflammatory monocyte population was sorted by FACS, centrifuged onto slides and stained with HE. Classical monocytes (left) and small Mikullicz cells (middle) were observed 3 days post-infection. Large Mikulicz cells (right) were present 5 days post infection. Scale bar: 10 µm.

To understand the immune process induced by *K. rhinoscleromatis* infection, we performed FACS analysis of the cell populations recruited to lungs of BALB/c mice infected with 2.10^7^ *K. rhinoscleromatis* or 2.10^4^ Kp52.145 at 1, 3 and 5 days post-infection (Fig 2A and Supporting Information Fig 2A). One day after infection, granulocytes (Gr1^+^ F4/80^−^ CD11b^+^ CD11c^−^) were recruited both during *K. rhinoscleromatis* or Kp52.145 infections as compared to control mice and represented 14 and 7% of the total cell number, respectively. While 5 days after *K. rhinoscleromatis* infection, granulocytes accounted for 38% of the cells, this population transiently increased during 3 days before returning to basal level 5 days after Kp52.145 infection. Both Kp52.145 and *K. rhinoscleromatis* induced a slight increase in the number of resident monocytes (Gr1^−^ F4/80^+^ CD11b^+^ CD11c^−^). Alveolar macrophages (Gr1^−^ F4/80^+^ CD11b^−^ CD11c^+^) were initially present in similar amounts in control, Kp52.145 and *K. rhinoscleromatis* infected mice, but interestingly, upon *K. rhinoscleromatis* infection this population had disappeared by day 3. Finally, inflammatory monocytes (Gr1^+^ F4/80^+^ CD11b^+^ CD11c^−^) were never observed in Kp52.145-infected or control mice. In contrast, and surprisingly, after *K. rhinoscleromatis* infection, lungs hold in 1.6 × 10^7^ and 3.3 × 10^7^ inflammatory monocytes 3 and 5 days post-infection, respectively, representing 15% of the whole lung cell population (Fig 2A and Supporting Information Fig 2A). To obtain further evidence of the inflammatory monocyte nature of these cells, we observed that they were Ly6C^+^ and Ly6G^−^ (Supporting Information Fig 3). We also noted that inflammatory monocytes presented a bigger size than resident monocytes as indicated by their forward side scatter profile (Fig 2B). We next assessed whether infection with *K. rhinoscleromatis* would develop similarly in C57BL/6 mice and therefore compared both the number of inflammatory monocytes and presence of Mikulicz cells in BALB/c and C57BL/6 mice. While 14.3% of total lungs cells were inflammatory monocytes in BALB/c mice, they represented only 7.9% in C57BL/6 mice (Supporting Information Fig 2B). Similarly, 1500 and 850 Mikulicz cells/mm^2^ were observed on histology sections in BALB/c and C57BL/6 mice, respectively (Supporting Information Fig 2C). Therefore, infection of mice with *K. rhinoscleromatis* is characterized by a strong recruitment of large inflammatory monocytes in two different mouse genetic backgrounds.

### Mikulicz cells are atypical inflammatory monocytes

Interestingly, we observed by histology that inflammatory monocytes recruitment was concomitant to Mikulicz cells appearance, suggesting that Mikulicz cells were inflammatory monocytes. To confirm this hypothesis, the granulocyte, resident monocyte and inflammatory monocyte populations were sorted by FACS and observed by microscopy 3 and 5 days post-infection. Cells presenting morphological features characteristic of Mikulicz cells, large cells with numerous vacuoles containing bacilli and a compressed nucleus, were exclusively found in the inflammatory monocyte population (Fig 2D). They were absent from the granulocyte and resident monocyte populations, demonstrating that Mikulicz cells were indeed inflammatory monocytes. Classical monocytes were also observed in the sorted inflammatory monocyte population 3 days post-infection. The quantification of the size of inflammatory monocytes during the infection (Fig 2C) revealed that 3 days post-infection 66% of the cells had a classical monocytes appearance and size of about 100 µm^2^, while small Mikulicz cells of 140–250 µm^2^ represented 44% of the inflammatory monocytes population. Five days after infection, 98% of inflammatory monocytes showed a Mikulicz cell phenotype and had an increased size ranging between 200 and 400 µm^2^ (Fig 2D). This observation suggested that, progressively, inflammatory monocytes phenotypically matured in large Mikulicz cells containing phagocytozed bacteria within enlarging vacuoles. Altogether, the morphology and cell size and the kinetics of appearance demonstrated that Mikulicz cells were indeed atypically matured inflammatory monocytes that accumulate in lungs during *K. rhinoscleromaris* infection.

### Recruitment of Mikulicz cells from the bone marrow is CCR2-independent

**Figure 3:**
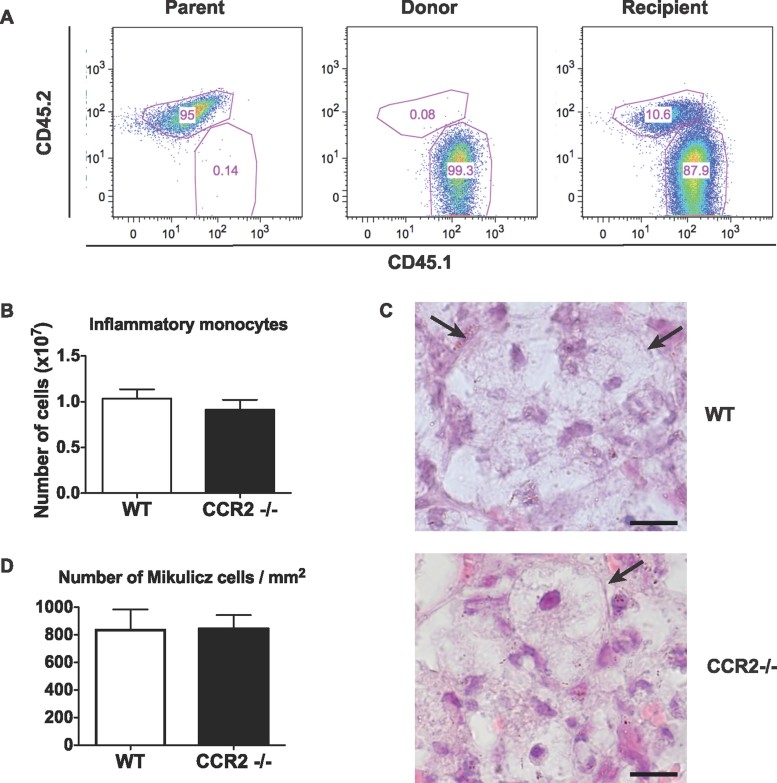
CCR2-independent recruitment of Mikulicz cells from the bone marrow to the lungs upon *K. rhinoscleromatis* infection. A. Expression of CD45.1 and CD45.2 markers on gated inflammatory monocytes from non-irradiated F1 (C57BL/6;BALB/c, parent), CD45.1-expressing C57BL/6 (donor) and irradiated and reconstituted F1 (recipient) infected with *K. rhinoscleromatis*. Graphs show representative data of one mouse out of ten infected recipient mice from two independent experiments. B. Number of inflammatory monocytes in lungs from CCR2^−/−^ and C57BL/6 WT mice 3 days post-infection with *K. rhinoscleromatis*. Data are mean ± SEM from 9 to 10 mice per group from two independent experiments. C. Typical Mikulicz cells are present in lungs from infected C57BL/6 WT or CCR2^−/−^ mice (arrows). Scale bar: 10 µm. D. Quantification of Mikulicz cells in lung sections of C57BL/6 WT or CCR2^−/−^ mice. Data are mean ± SEM. *n* = 10.

Inflammatory monocytes are known to be recruited from the bone marrow to inflamed tissues (Shi & Pamer, 2011). To check if it was the case in the context of pulmonary *K. rhinoscleromatis* infection, we designed an experiment where mice carrying the CD45.2 allele were reconstituted with bone marrow isolated from mice congenic for the CD45.1 marker. Because the results were more pronounced in a BALB/c background, and mice carrying the CD45.1 marker were in a C57BL/6 background, we generated C57BL/6;BALB/c chimera expressing the CD45.2 allele. These mice were sub-lethally irradiated and reconstituted with bone marrow cells isolated from C57BL/6 mice congenic for the CD45.1 marker. Mice were then infected 6 weeks later and inflammatory monocytes were identified by FACS and checked for their expression of CD45.1 or CD45.2 markers. As expected, inflammatory monocytes from both types of controls were carrying the markers CD45.2 (Fig 3 Parent) or CD45.1 (Fig 3 Donor), respectively. In irradiated and reconstituted mice, inflammatory monocytes carried the donor CD45.1 marker showing that inflammatory monocytes had been recruited to the lungs from bone marrow precursors.

Expression of the CCR2 receptor by inflammatory monocytes mediates their egress from the bone marrow and is required in several infectious models (Serbina et al, 2008; Shi & Pamer, 2011). Hence, we tested whether CCR2 was involved in the recruitment of Mikulicz cells. C57BL/6 CCR2 ^−/−^ mice were infected with *K. rhinoscleromatis* and the bacterial load and the presence of inflammatory monocytes/Mikulicz cells assessed by FACS and histology 1, 3 and 5 days post-infection. Unexpectedly, the disease progressed normally in CCR2-deficient mice as compared to wild-type (WT) mice. No obvious change in the number of bacteria present in the lungs was observed (Supporting Information Fig 4). Resident monocytes, alveolar macrophages, granulocytes (Supporting Information Fig 4) and more importantly, inflammatory monocytes were detected in similar amounts in CCR2^−/−^ and WT mice (Fig 3B and Supporting Information Fig 4). Mikulicz cells were observed by histology (Fig 3C) in both WT and CCR2^−/−^ mice in similar amount (Fig 3D). These results indicated that the recruitment of inflammatory monocytes from the bone marrow to the lungs and their subsequent acquisition of a Mikulicz cells phenotype is independent of CCR2 during *K. rhinoscleromatis* infection.

### IL-10 is highly increased after *K. rhinoscleromatis* infection

**Figure 4:**
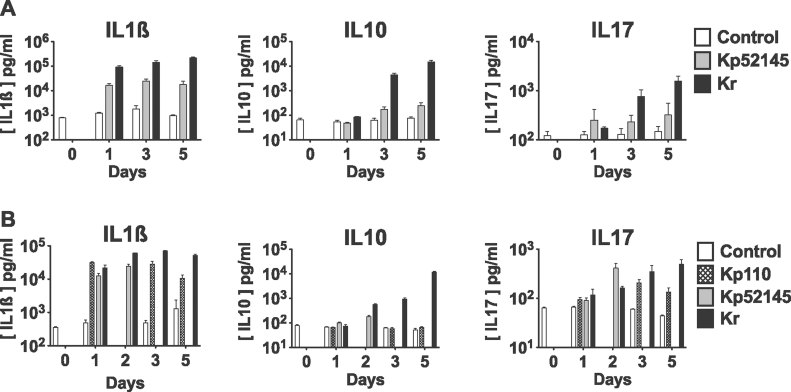
Production of IL-1β, IL-10 and IL-17 in the lungs of mice infected with *K. rhinoscleromatis*, Kp52.145 or Kp110. A. BALB/c mice were injected with saline or infected with 2.10^7^ *K. rhinoscleromatis* or 2.10^4^ Kp52.145 for 1, 3 or 5 days. Lungs were homogenized and cytokines were measured in the extracts by ELISA. Data are mean ± SEM from 8 to 15 mice from two independent experiments. B. Mice were injected with saline or infected with 2.10^7^ *K. rhinoscleromatis*, Kp52.145 or Kp110 strains. Cytokines were measured at different days post infection (1, 2, 3 and 5 for *K. rhinoscleromatis*; 1, 3 and 5 for Kp110; 1 and 2 for Kp52.145). Data are mean ± SEM from 3 to 9 mice from two independent experiments.

Cytokines are key mediators of cell recruitment and maturation during an immune response and therefore distinctive of the type of immune response. Strikingly, the histological analysis revealed an absence of destructive lesions around Mikulicz cells suggesting that *K. rhinoscleromatis* was able to dampen the inflammatory response, likely through the induction of a specific set of cytokines. Hence, we characterized the cytokine profile during *K. rhinoscleromatis* infection by measuring the production of some of them in mice lung extracts. When BALB/c mice were infected with either 2.10^7^ *K. rhinoscleromatis* or 2.10^4^ Kp52.145, the pro-inflammatory cytokines IL-1β, IL-6, IL-17, were produced in high amounts from 1 day post-infection onwards (Fig 4A and Supporting Information Fig 5). Importantly, we observed that the anti-inflammatory cytokine IL-10 was highly produced after *K. rhinoscleromatis* infection, up to 61 times more than after Kp52.145 infection 5 days post-infection.

To establish that the lower cytokine expression profile observed with Kp52.145 was not due to the lower inoculum of Kp52.145 as compared to *K. rhinoscleromatis*, we measured cytokines expression in animals infected with the same inoculum (2.10^7^) of Kp52.145, *K. rhinoscleromatis* or Kp110. The latter strain is an avirulent plasmid-cured derivative of Kp52.145. In contrast to Kp52.145, which kills mice after 2 days, Kp110 allows to follow the kinetics of cytokine expression during 5 days. IL-1β and IL-17 were expressed in similar amounts in mice infected with *K. rhinoscleromatis*, Kp52.145 or Kp110 the first 3 days of infection and then decreased at day 5 in Kp110-infection (Fig 4B) because the bacteria were being cleared from the organ (Supporting Information Fig 6). Interestingly, we observed that IL-10 was still highly expressed after *K. rhinoscleromatis* infection, contrasting sharply with the basal expression level observed during the 5 days post-infection after Kp110 infection. Altogether, these observations suggested that, while classical pro-inflammatory cytokines were produced during both Kp52.145 and *K. rhinoscleromatis* infection, the *K. rhinoscleromatis*-specific IL-10 secretion played an important role in the establishment of a proper environment for the recruitment and maturation of Mikulicz cells.

### IL-10 is essential to the occurrence of Mikulicz cells

**Figure 5:**
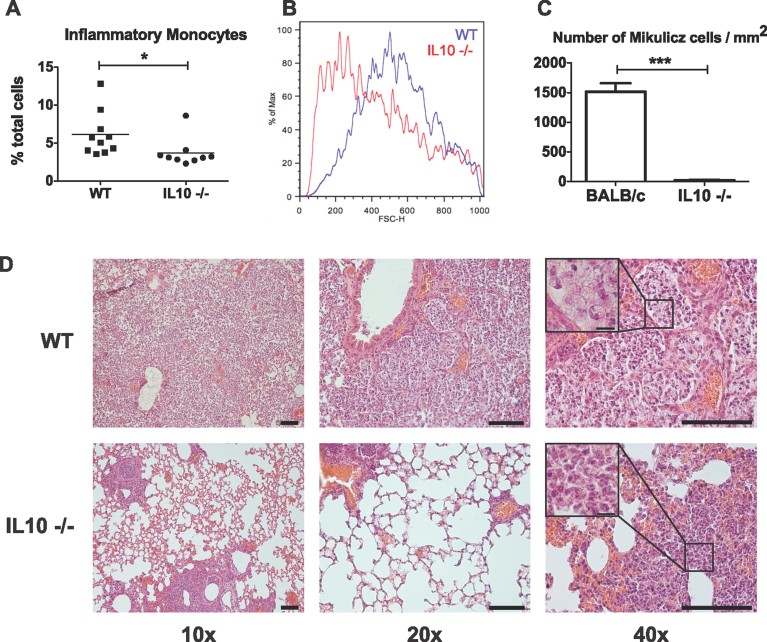
Absence of Mikulicz cells in IL10^−/−^ mice. A. Percentage of inflammatory monocytes in lungs from IL-10^−/−^ and BALB/c WT mice 3 days post-infection with 10^6^ *K. rhinoscleromatis*. (*p = 0.04). Data are representative of 7 to 10 mice from three independent experiments. B. Forward scatter histogram of inflammatory monocytes in WT (blue) and IL10^−/−^ (red) mice. Cells are smaller in IL10^−/−^ mice. C. Comparison of number of Mikulicz cells in tissue sections between BALB/c WT and IL10^−/−^ mice. Data are mean ± SEM. *n* = 16 and 38. (***p < 0.0001). D. Histology, representative examples of lungs in IL10^−/−^ and BALB/c WT mice. Inset show Mikulicz cells in WT and granulocytes and polymorphonuclear cells in IL10^−/−^ mice. Scale bars 100 µm, inset 10 µm.

To evaluate the putative role of IL-10 in the pathogenesis of rhinoscleroma we challenged BALB/c IL-10-deficient mice with *K. rhinoscleromatis* and compared them to WT BALB/c mice. As IL-10^−/−^ mice infected with 2.10^7^ *K. rhinoscleromatis* were highly susceptible to the infection and died within 3 days, mice were infected with a lower inoculum (10^6^ *K. rhinoscleromatis*) and after 3 days, lungs were collected to perform FACS analysis and histology. We observed that the percentage and total number of inflammatory monocytes after infection was reduced by around 40% in IL-10^−/−^ mice when compared to the WT (Fig 5A and Supporting Information Fig 7A). This indicated that although IL-10 plays a possible role in the recruitment of inflammatory monocytes, it is not essential for this process. However, as we still observed the recruitment of these cells after *K. rhinoscleromatis* infection, we wondered whether inflammatory monocytes presented the typical features of Mikulicz cells, in particular their classical large size. By analyzing the forward scatter parameter, we observed that there was a remarkable difference in the size of the cells (Fig 5B). The inflammatory monocytes present in the lungs of IL-10^−/−^ mice were smaller compared to those found in WT mice, a suggestion that they likely were normal inflammatory monocytes or immature Mikulicz cells. Histological analysis of the infected lungs specified further this observation (Fig 5C). Mikulicz cells were present at an average density of 1500 cells/mm^2^ of tissue (Fig 5D), filling the alveoli in WT mice. On the contrary their number was dramatically reduced to 20 cells/mm^2^ in IL-10^−/−^ mice, which instead presented a dense peribronchial inflammatory infiltrate containing numerous polymorphonuclear cells. These data suggested that while IL-10 contributed to the recruitment of inflammatory monocytes to the site of infection, it is essential to their phenotypic maturation into Mikulicz cells.

**Figure 6:**
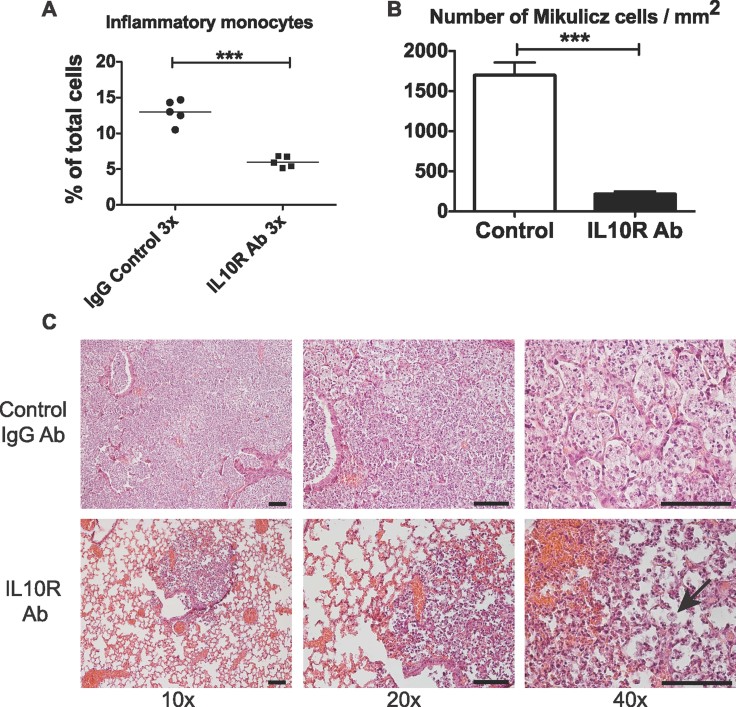
Blocking IL-10R leads to the absence of Mikulicz cells. A. Percentage of inflammatory monocytes in lungs of BALB/c mice treated with IL-10R blocking antibody or control IgG 4 days post-infection with *K. rhinoscleromatis*. (***p < 0.001). Data are representative of 10 mice from two independent experiments. B. Comparison of number of Mikulicz cells in tissue sections between BALB/c WT mice and mice injected with IL10R neutralizing antibody. Data are mean ± SEM. *n* = 39 and 40. ***p < 0.0001. C. Histology, representative examples of lungs in mice injected with IL-10R neutralizing antibody or control IgG. Arrow points to a rare Mikulicz cell observed. Scale bars: 100 µm.

To further support this observation, we used a pharmacological approach to block IL-10 receptor and evaluate in this context, inflammatory monocytes and Mikulicz cells presence. BALB/c mice were infected with 2.10^7^ *K. rhinoscleromatis* and then injected with 100 µg of anti-IL-10R mAb or isotype-matched control IgG at days 1, 2 and 3. By FACS, IL-10R blockade resulted in a sharp decrease in the percentage and number of inflammatory monocytes in the lungs by more than 50% compared to control mice (Fig 6A and Supporting Information Fig 7B). By histology, we observed a drastic reduction in the number of Mikulicz cells present in the infected lungs of anti-IL-10R treated mice (Fig 6B and C). Mikulicz cells were present at an average density of 1700 and 215 cells/mm^2^ in control mice and IL10R-treated mice, respectively. These data further validate the importance of IL-10 in the formation of Mikulicz cells. Collectively, these data suggest that IL-10 signalling is important for inflammatory monocytes recruitment and their maturation into Mikulicz cells.

## DISCUSSION

Even though rhinoscleroma is a rare disease, cases are now being reported in previously rhinoscleroma-free regions (Botelho-Nevers et al, 2007). The infection can lead to severe respiratory impairment and nasal deformities. Current therapies rely on long cumbersome antibiotics treatment and/or surgery and there is a tendency for recurrence in endemic areas. The etiologic agent of rhinoscleroma, *K. rhinoscleromatis* is very closely related to *K. pneumoniae*, both species presenting approximately 99.5% of homology (Brisse et al, 2009). Nevertheless they cause very different diseases. While *K. pneumoniae* can cause acute destructive inflammation of the lung parenchyma, the first steps of infection with *K. rhinoscleromatis* are comparable but then a switch to a less inflammatory profile is observed. Yet, the molecular and cellular mechanisms underlying rhinoscleroma are not yet understood. In this study, using several congenic and knock-out mice strains, we reinvigorated the initial work of Steffen and Smith (1961) and successfully developed and characterized a murine model reproducing the earliest signature of rhinoscleroma: the appearance of the characteristic Mikulicz cells in the respiratory tract. Mikulicz cells are often described morphologically as foamy macrophages. Foamy macrophages are commonly found in several chronic diseases such as atherosclerosis or persisting infections such as tuberculosis, leprosy, or infection caused by *Chlamydia pneumoniae* or *T. gondii*. They are notably characterized by their numerous lipid-containing vesicles that result from alteration in the transport of lipids (Russell et al, 2009). These lipid vesicles that are easily observed by electron microscopy are never seen in Mikulicz cells. In the case of tuberculosis, foamy macrophages have been shown to express markers characteristic of dendritic cells (Ordway et al, 2005). However, these macrophages differ remarkably from Mikulicz cells that have numerous bacteria-containing vacuoles that are believed to arise following increased osmotic pressure due to the presence within the vacuoles of bacterial capsular polysaccharide (Hoffmann et al, 1973). Mikulicz cells studied here are thus a specific feature of infection with *K. rhinoscleromatis*. We also characterized the nature of these cells and the host immunological environment leading to their maturation in the lung alveolar space: *K. rhinoscleromatis*-elicited Mikulicz cells were shown to carry markers identifying them as inflammatory monocytes; the comparison between cytokines expression profiles in *K. pneumoniae*- and *K. rhinoscleromatis*-infected lungs showed the specific expression of IL-10 concomitantly to the appearance of Mikulicz cells; and IL-10 was eventually shown to be crucial for the maturation of Mikulicz cells. Altogether this model represents a unique model of cellular microbiology to study the transition between an acute to a chronic infectious state.

To our knowledge, three other models of rhinoscleroma were previously developed in rabbit, rat and mouse (Gaafar et al, 2000; Steffen & Smith, 1961; Talaat et al, 1978). Despite different experimental conditions (infectious doses, inoculation route, time after infection), a common feature of all models is the occurrence of Mikulicz cells, confirming the pivotal function of these cells in the infectious process. However, granuloma formation is only observed in rabbits and rats upon repeated infections. This feature of the disease was not considered here in mice, as we wished to analyze the very early steps of infection and the moment where both *K. pneumoniae* and *K. rhinoscleromatis* infection diverge. Our model, which is very well suited to study the early step of infection, will also need to be further evaluated for its capacity to generate chronic (*i.e.* granulomatous) lesions as well as translated to human samples, although the paucity of such samples makes this a difficult task.

Thanks to our *in vivo* model, we characterized for the first time, the host immunological environment allowing the maturation of Mikulicz cells. Importantly, these cells carried markers of inflammatory monocytes and were observed specifically in mice lung parenchymal tissue upon infection with *K. rhinoscleromatis* but never after Kp52.145 or Kp110 infection at both high and low inocula. Inflammatory monocytes are a subset of monocytes circulating in the bloodstream that are recruited to inflammation sites. They are essential for defense against several bacterial, parasite and fungi infections (reviewed in Serbina et al, 2008; Shi & Pamer, 2011) such as *L. monocytogenes* (Kurihara et al, 1997), *Mycobacterium tuberculosis* (Peters et al, 2004), *S. typhimurium* (Rydström & Wick, 2007), *S. pneumoniae* (Winter et al, 2009), *Yersinia pestis* (Ye et al, 2011), *Burkholderia mallei* (Goodyear et al, 2010), *T. gondii* (Dunay et al, 2008; Robben et al, 2005), *Aspergillus nidulans* (Blease et al, 2000; 2001) or *Cryptococcus neoformans* (Osterholzer et al, 2009). They are recruited from the bone marrow in a CCR2-dependent manner (Serbina & Pamer, 2006; Tsou et al, 2007). Here we showed that upon *K. rhinoscleromatis* infection, inflammatory monocytes were recruited to the lungs, and that, surprisingly, this did not require CCR2. We actually observed that CCL2/MCP1, the main ligand of CCR2, was highly expressed during both infections with *K. rhinoscleromatis* and *K. pneumoniae*, suggesting that CCL2/MCP1 signalling on CCR2 does not play a major role in the *K. rhinoscleromatis*-specific inflammatory monocytes recruitment in this particular disease (Supporting Information Fig 5). Moreover, it has been recently shown that the spleen hosts a pool of monocytes that can be recruited to inflammatory sites (Swirski et al, 2009). We have tested this possibility and found Mikulicz cells in the lungs from splenectomized mice indicating they do not originate from splenic monocytes (Supporting Information Fig 8). It is therefore likely that Mikulicz cells recruitment relies on multiple chemokines signalling. Indeed, recent reports have shown that inflammatory monocytes recruitment to the brain or liver during *L. monocytogenes* infection is CCR2-independent (Drevets et al, 2010; Serbina & Pamer, 2006; Shi et al, 2010). In addition, during atherosclerosis, recruitment of monocytes to atherosclerotic plaque relies on CCR2, CX3CL1/CX3CR1 and CCR5 (Saederup et al, 2008; Tacke et al, 2007), showing that multiple receptors can be used to recruit these cells to inflammation sites.

Another striking observation was the complete disappearance of alveolar macrophages upon *K. rhinoscleromatis* infection. This is likely explained by their rapid death shortly after infection as this is also observed during *S. pneumoniae* infection (Dockrell et al, 2003). As alveolar macrophages are important cells regulating the initiation of inflammation in the lungs, replacement of alveolar macrophages by recruited monocytes may have profound effects on pathogenesis.

The cytokines expression profile between *K. rhinoscleromatis* and *K. pneumoniae* infections shows minor differences in expression of IL-1β, IL-6 and IL-17, when similar infection kinetics is used. Indeed, when similar infectious doses are applied, although infection with virulent Kp52.145 is lethal, the cytokines IL-1β, IL-6, and IL-17 and the chemokines CCL2, CCL3 and CCL4 are produced in similar manner. One can thus deduce that the production of these pro-inflammatory cytokines and chemokines results more from the infection dose than specific induction by the pathogen and is a global signature of the infection. However, one of the main features of *K. rhinoscleromatis* infection was the strong expression of IL-10, an anti-inflammatory cytokine with a crucial role in limiting the immune response during infection to pathogens and thereby preventing damage to the host (Moore et al, 2001; Saraiva & O'Garra, 2010). Decreased or delayed IL-10 production results in severe tissue damage, a consequence of an uncontrolled inflammatory response, in a number of infections including *T. gondii* (Gazzinelli et al, 1996), *Plasmodium* spp. (Couper et al, 2008b; Li et al, 1999), and *Trypanosoma cruzi* (Hunter et al, 1997). This was also similar to *K. rhinoscleromatis* infection as we have observed that IL-10 deficient mice were not capable of recruiting Mikulicz cells and showed dense and highly inflammatory lesions. In contrast, in other infections such as with *K. pneumoniae*, experimental inhibition of IL-10 signalling restores pathogen control and reduces the severity of disease (Greenberger et al, 1995). What causes such a different outcome during infection with *K. rhinoscleromatis* and *K. pneumoniae* remains to be investigated. Strikingly, in two experimental conditions, using knockout mice or injection of blocking antibody, we observed a significant reduction in the number of Mikulicz cells. This suggests an important role of IL10 either in controlling the maturation of inflammatory monocytes in Mikulicz cells or the establishment of a proper environment for their occurence. The source of IL-10 in lungs infected with *K. rhinoscleromatis* is not yet known. Although lymphocytes, mast cells, bronchial epithelial cells (Bonfield et al, 1995) and type I pneumocytes (Haase et al, 2007) produce IL-10, monocytes and macrophages, and polymorphonuclear cells have been identified as its major source during a variety of infections (Couper et al, 2008a; Cyktor & Turner, 2011), and microbes have evolved mechanisms by which they subvert production of IL-10. As the kinetic and production of IL-10 matched the recruitment of Mikulicz cells, it is possible that these cells are the IL-10 producers. Altogether, these observations point to a major role of IL-10 in the recruitment of inflammatory monocytes and their phenotypic maturation into Mikulicz cells during *K. rhinoscleromatis* infection.

In this study, we have successfully identified IL-10 as a single crucial factor for the occurrence of Mikulicz cells. The highly vacuolated characteristic of Mikulicz cells has not been reported for inflammatory monocytes infected with other pathogens. Mikulicz cells thus represent a peculiar state of inflammatory monocytes unable to digest bacteria. We observed that absence of Mikulicz cells in IL10^−/−^ mice correlated with a decreased bacterial load (Supporting Information Fig 7C) suggesting that Mikulicz cells could represent a protective or proliferating niche for the bacteria and be involved in the persistence of the disease (Canalis & Zamboni, 2001). However, this observation was not reproduced in anti-IL10R-treated mice (data not shown), although the reason for such discrepancy is unknown. Nevertheless, our recent study of the genetic diversity of *K. pneumoniae* subspecies showed a restricted pattern of metabolic activities for *K. rhinoscleromatis* (Brisse et al, 2009). This observation indicates that this bacterium might have evolved towards adaptation to specific host conditions and it suggests that its persistence involves intracellular survival in Mikulicz cells and consequently the development of chronic inflammation.

In summary, *K. rhinoscleromatis* induced the appearance of Mikulicz cells in lungs of infected mice. These cells presented the same morphological characteristics as those found in the human disease. For the first time, we demonstrated Mikulicz cells to be atypical inflammatory monocytes whose maturation is dependent on IL-10. This disease represents a new interesting model to further understand the control and balance of inflammation by pathogens and their ability to induce acute *versus* chronic infection. Moreover, a detailed understanding of how IL-10 expression is regulated during rhinoscleroma may unravel new therapeutic strategies to modulate IL-10 production.

## MATERIALS AND METHODS

### Ethics statement

The study was carried out in accordance with the French and Europeans regulations on care and protection of the laboratory animals (EC Directive 86/609, French Law 2001-486 issued in June 2001). Mice were housed under standard conditions of feeding, light and temperature with free access to food and water. Protocols were approved by the Institut Pasteur animal care and use committee (protocol 05-59) and the Direction des Services Vétérinaire de Paris (permit 75-713 to RT).

### Bacterial strains and culture conditions

The *K. pneumoniae* subsp. *rhinoscleromatis* SB3432 strain was isolated in 2004 at the Avicenne hospital, Bobigny, France from a biopsy of the left nasal cavity of an 11-years old patient diagnosed with rhinoscleroma. Its biochemical characteristics and MLST typing have been described elsewhere (Brisse et al, 2009). The *K. pneumoniae* subsp. *pneumoniae* Kp52.145 strain is a highly virulent strain in mice (Riottot et al, 1981) carrying the large pKP100 virulence plasmid (Nassif & Sansonetti, 1986). The *K. pneumoniae* subsp. *pneumoniae* Kp110 strain is a pKP100-cured derivative of Kp52.145 and is avirulent in mice (Nassif et al, 1989). Inocula were prepared from bacteria grown overnight on LB or TCS plates at 37°C resuspended in physiological saline.

### Infection of mice

BALB/cJ and C57BL/6 mice were purchased from Janvier (Le Genest St. Isle, France). CCR2^−/−^ mice (C57BL/6 background, Boring 1997) were obtained from the Jackson Laboratory (Bar harbor, Maine, USA). BALB/c IL-10^−/−^ mice were kindly provided by Anne O'Garra (IMRC, London) and have been previously described (Gazzinelli et al, 1996). Six to eight weeks-old mice were anesthetized with acepromazine (Calmivet, 1.5 mg/kg, Vetoquinol) and ketamine (Imalgene, 31.25 mg/kg, Merial) and then infected intranasally with 20 µl bacterial suspension. Control animals received physiological saline. At various times post-infection mice were euthanized by i.p. injection of sodium pentobarbital (Dolethal, 600 mg/kg, Vetoquinol). Mouse groups were rigorously age- and sex-matched for each infection experiment.

**The paper explained**

**PROBLEM**

Rhinosleroma is a rare and underestimated chronic infection of the upper airways in humans, which is caused by the bacterium *K. pneumoniae* subspecies *rhinoscleromatis*. *K. rhinoscleromatis* is very closely related to *K. pneumoniae* subspecies *pneumoniae*, but causes a very different disease. Rhinoscleroma is characterized by the occurrence of granuloma encompassing large atypical phagocytic cells containing ingested bacteria called Mikulicz cells. However, the mechanisms leading to the development of this disease are poorly understood, nor what differentiate the host response in *K. rhinoscleromatis* and *K. pneumoniae* infections.

**RESULTS**

We developed a new model of rhinoscleroma recapitulating the formation of Mikulicz cells and investigated the nature of these cells and the factors responsible of their appearance. We found that Mikulicz cells are a subset of recruited phagocytes identified as inflammatory monocytes, recruited specifically upon *K. rhinoscleromatis* infection. These cells are recruited independently from CCR2. Both *K. pneumoanie* and *K. rhinoscleromatis* infections are characterized by the production of classical inflammatory cytokines. However, *K. rhinoscleromatis* infection is characterized by the intense production of interleukin-10, an anti-inflammatory cytokine. While IL-10 plays a role in the recruitment of inflammatory monocytes, it is necessary for their maturation in Mikulicz cells.

**IMPACT**

Our findings provide the first cellular and molecular characterization of rhinoscleroma and show the important role of IL-10 in the phenotypic maturation of Mikulicz cells, while starting to highlight some fundamental differences between *K. pneumoniae* and *K. rhinoscleromatis* pathogenesis. Further research is required to better understand the role of Mikulicz cells in the infectious process and what specifically differentiate *K. pneumoniae* from *K. rhinoscleromatis*.

### Determination of infectious doses and CFUs

The infectious dose of *K. rhinoscleromatis* required to reproduce the appearance of Mikulicz cells was determined 7 days post-infection by histological observation of mice lungs infected with 1.10^4^ to 1.10^8^ CFU/mouse. Two mice per dose were analyzed. The dose of 2.10^7^ bacteria was used for strains *K. rhinoscleromatis* and Kp110. Mice infected with 2.10^7^ Kp52.145 did not survive for longer than 2 days. In order to compare the physiological effects of *K. rhinoscleromatis* and Kp52.145 isolates with the same kinetics, mice were thus infected with 2.10^4^ Kp52.145. CFUs were determined by plating serial dilutions of lung homogenates in 3 ml ice-cold 10 mM HEPES buffer supplemented with 0.2 mM EDTA and 0.1% bovine serum albumin.

### Histology and microscopy

At various times post-infection lungs were inflated with 4% buffered paraformaldehyde (PFA) and fixed overnight at 4°C. Paraffin-embedded tissue blocks were cut into 5–7 µm sections and stained with hematoxylin–eosin (HE). Images were acquired on a Nikon Eclipse E800 microscope equipped with a Nikon DXM100F camera using the Nikon EclipseNet software. Lenses used were 10× NA 0.45, 20× NA 0.75, 40× or 100× NA 1.4. Levels were adjusted on the entire images using Photoshop. Quantification of Mikulicz cell number on lung sections was done using the 40× objective and by counting the number of Mikulicz cells in 6–10 different fields per histological sections.

For transmission electron microscopy, tissues were fixed overnight with 2.5% glutaraldehyde and 2% PFA in 0.08 M cacodylate buffer stained with 1% osmium tetroxyde for 2 h and embedded in EPON resin mixture. If indicated, 0.75% ruthenium red was added during the fixation step. Ultra thin sections were examined with a JEOL 1200EX II electron microscope at 80 kV.

### Generation of bone marrow chimeric mice

C57BL/6 males were crossed with BALB/c females to generate BALB/c;C57BL/6 F1 mice. Both parent strains were carrying the CD45.2 (Ly5.2) allele. In reconstitution experiment, F1 mice were used as recipient and C57BL/6 mice carrying the CD45.1 allele were used as donors. Eight-weeks-old F1 mice were sub-lethally irradiated (950 rad) and reconstituted with 5 × 10^6^ bone marrow cells from CD45.1 mice injected in the retro-orbital sinus. Mice were then infected with *K. rhinoscleromatis* 6 weeks after reconstitution.

### Lung cells isolation

At various time post-infection the five pulmonary lobes were aseptically removed, cut into small pieces and incubated on ice in 3 ml buffer (10 mM HEPES supplemented with 0.2 mM EDTA and 0.1% BSA) for 20 min. They were then fixed for 1 h by adding 3 ml of 4% PFA v/v. Cells were isolated after filtration through a 100 µm mesh. Red blood cells were eliminated by incubating samples for 1 min at room temperature in a red blood cell lysis buffer (Sigma–Aldrich). Cells were washed twice with buffer and kept at 4°C before immunological staining.

### Flow cytometry analysis and sorting

Each step was performed on 5 × 10^6^ cells in a final volume of 100 µl. Isolated cells were blocked with 10 µg/ml Mouse BD Fc Block (BD Biosciences) at 4°C for 20 min, washed twice with the supplemented HEPES buffer and labeled with the following antibodies: anti-Gr1-FITC (clone RB6-8C5, 1/200 dilution, BD Pharmigen), anti-CD11b-PERCP-Cy5.5 (clone M1/70, 1/200 dilution, BD Pharmingen), anti-F4/80-PE (clone BM8, 1/100 dilution, eBiosciences) and anti-CD11c-APC (clone N418, 1/300 dilution, eBioscience). Data were collected using a FACSCalibur or Cyan cytometers (Becton Dickinson, Sunnyvale, CA) and analyzed with FlowJo software (Tree Star Inc., Ashland, OR) to visualize granulocytes (Gr1^+^ F4/80^−^ CD11b^+^ CD11c^−^), resident monocytes (Gr1^−^ F4/80^+^ CD11b^+^ CD11c^−^), alveolar macrophages (Gr1^−^ F4/80^+^ CD11b^−^ CD11c^+^) or inflammatory monocytes (Gr1^+^ F4/80^+^ CD11b^+^ CD11c^−^). Mice were inoculated with saline as control, with *K. rhinoscleromatis* or Kp52.145. For cell sorting, cells were collected and stained as described above, and sorted using a FACSARIA cell sorter (BD Biosciences). For morphologic analysis, FACS-sorted cell populations were cytospun onto microscope slides at 30 g for 5 min and stained with HE. Quantification of cell surface of the sorted cells was performed using AxioVision 4.5 software from Zeiss.

### Cytokine/chemokine quantification by ELISA

At various time post-infection the five pulmonary lobes were removed and crushed in 2.5 ml of ice cold PBS-0.5% TritonX-100 containing EDTA-free protease inhibitor (Roche). Twenty microlitres were removed to determine the number of CFU/lung. Samples were centrifuged at 14,000 × *g* for 5 min, the supernatants frozen in liquid nitrogen and stored at −80°C. The following cytokines/chemokines were measured: IL-1β, IL-6, IL-10, IL-17, CCL2, CCL3 and CCL4 (Duoset, all from R&D Systems). Assays were performed according to the manufacturer's recommendations.

### *In vivo* monoclonal antibody treatment

For IL-10R blocking experiments, mice were injected i.p. with 100 µg of anti-mouse IL-10R (clone 1B1.3a, BD Pharmigen) or isotype matched IgG1 (clone R3-34, BD Pharmigen) antibodies in 100 µl PBS. Antibodies were administrated on days 1, 2 and 3 after *K. rhinoscleromatis* infection.

### Statistical analysis

Statistical analysis were performed with Prism 5 (Graph Pad) using one way ANOVA, Student *t*-test or Mann–Whitney tests when appropriate. Differences were considered statistically significant with *p* < 0.05 (*p < 0.05; **p < 0.01; ***p < 0.001).

## Author contributions

CF, ASA, AC, SB, PJS and RT conceived and designed experiments; CF, ASA, ST, TP, MCP, AK and RT performed experiments, CF, ASA, ST, TP, MH, AK, SB, PJS and RT analyzed the data, CF, ASA, and RT wrote the manuscript; TP, MH, AC, SB and PJS gave comments on the manuscript.

## Notes

As a service to our authors and readers, this journal provides supporting information supplied by the authors. Such materials are peer reviewed and may be re-organized for online delivery, but are not copy-edited or typeset. Technical support issues arising from supporting information (other than missing files) should be addressed to the authors.
