# Supplementary material for: A novel murine model of rhinoscleroma identifies Mikulicz cells, the disease signature, as IL-10 dependent derivatives of inflammatory monocytes
Source: EMBO Mol Med. 2013 Apr 2;5(4):516–30. doi: 10.1002/emmm.201202023 (PMC3628109; doi:10.1002/emmm.201202023)
Supplement: Supplementary file 1 [file emmm0005-0516-sd1.pdf]

EMBO Molecular Medicine

A novel murine model of rhinoscleroma identifies Mickulicz cells, the disease signature, as IL-10 dependent derivatives of inflammatory monocytes

Cindy Fevre, Ana S. Almeida, Solenne Taront, Thierry Pedron, Michel Huerre, Marie-Christine Prevost, Aurélie Kieusseian, Ana Cumano, Sylvain Brisse, Philippe J. Sansonetti and Régis Tournebize

Corresponding author: Regis Tournebize, Institut Pasteur

Review timeline:

Submission date:	14 September 2012
Editorial Decision:	13 November 2012
Revision received:	31 January 2013
Accepted:	14 February 2013

Transaction Report:

(Note: With the exception of the correction of typographical or spelling errors that could be a source of ambiguity, letters and reports are not edited. The original formatting of letters and referee reports may not be reflected in this compilation.)

Editor: Natascha Bushati / Céline Carret

1st Editorial Decision 13 November 2012

Thank you for the submission of your manuscript to EMBO Molecular Medicine. We have now heard back two of the three referees whom we asked to evaluate your manuscript. To save you from any unnecessary loss of time, I am making a decision on your manuscript based on the two reports we have received.

As you will see from the reports below, the referees find the topic of your study of potential interest. However, they raise substantial concerns on your work, which should be convincingly addressed in a major revision of the present manuscript.

In particular, reviewer #2 highlights that additional data addressing a possible Mikulicz cell phenotype of non-inflammatory monocyte populations are required to strengthen the study. This referee would also like to see further quantification of Mikulicz cells in the histological data. In addition, the referee has concerns regarding the interpretation of the data demonstrating a role for IL-10.

Reviewer #3 is concerned that the functional properties of Mikulicz cells have not been analysed. This point has also been raised by referee #2 (point 6), and we would strongly encourage you to address it with further experiments. In addition, referee # 3 also raises concerns regarding the IL-10 data and we would suggest to further substantiate these findings by investigating whether recombinant IL-10 rescues the effect.

Given the balance of these evaluations, we feel that we can consider a revision of your manuscript if

you can convincingly address the issues that have been raised within the space and time constraints outlined below.

On a more editorial note, EMBO Molecular Medicine requires:

- The description of all reported data that includes statistical testing must state the name of the statistical test used to generate error bars and P values, the number (n) of independent experiments underlying each data point (not replicate measures of one sample), and the actual P value for each test (not merely 'significant' or 'P < 0.05') (please see http://onlinelibrary.wiley.com/journal/10.1002/(ISSN)1757-4684/homepage/ForAuthors.html#data2 for more information).

- Please also include a Table of Contents as the first page of your Supporting Information.

Revised manuscripts should be submitted within three months of a request for revision. If your revision will have to exceed this time frame, please contact the editor. Please also contact the editor as soon as possible if similar work is published elsewhere.

***** Reviewer's comments *****

Referee #2 (Comments on Novelty/Model System):

This is an important study, but is at times confusing and in requires much more clarification on the issues raised below. In particular, the analysis of the role of IL-10 is very confusing.

Referee #2 (General Remarks):

Comments on: A novel murine model of rhinoscleroma identifies Mickulicz cells, the disease signature, as IL-10 dependent derivatives of inflammatory monocytes

In this manuscript, Fevre and colleagues characterize a murine model of rhinoscleroma by infection with K. pneumoniae. A search of Pubmed indicates that there are no recent studies of rhinoscleroma in an animal model. In this regard, the authors have taken an important first step in describing a novel murine model of this infection, which will be important for understanding the mechanisms of disease pathogenesis.
The manuscript could be improved by considering the following:
1. With the data presented, the authors do not definitively show that Mikulicz cells are exclusively inflammatory monocytes. For example, in Figure 2, the authors sort inflammatory monocytes and show that these cells have a Mikulicz cell phenotype, which does not rule out that cells expressing other (non-inflammatory monocyte) markers could have this phenotype as well. Have the authors sorted and done cytospins on the other cell populations described? Also, it is not clear from the data shown whether 100% of the inflammatory monocytes were Mikulicz cells or whether the Mikulicz cells were a subpopulation of total inflammatory monocytes. This is important, as the phrases "inflammatory monocytes" and "Mikulicz cells" are used interchangeably throughout the manuscript.
2. The FACS data shown in figure 3B would have more impact if the authors used the percentages to calculate the total numbers of inflammatory monocytes. For example, if the CCR2-/- mice have fewer total lung cell numbers, then there would be fewer total numbers of inflammatory monocytes. It is necessary to show total cell numbers to conclude that CCR2 is not involved in the recruitment of these cells. Figure 3C could be improved by showing slides of both the wild type and CCR2-/- ; it would also be useful to quantify the numbers of Mikulicz cells by histology to confirm the phenotype shown by FACS and more conclusively exclude the involvement of CCR2 in their recruitment.
3. Can the authors add a representative histology image in Fig 3C of WT lungs, showing the presence of a similar frequency of Mikulicz cells.
4. There is only a subtle difference in the percentage of inflammatory monocytes shown in Figure 5. Fascinatingly, from the histological images, it appears that IL-10-/- mice have much fewer inflammatory cells. If total numbers of inflammatory monocytes were shown (by calculating from

the total number of lung cells), would there be a greater difference between wild type and IL-10-/- mice?
5. The interpretation for role of IL-10 in this model was very confusing. Despite there appearing to be fewer inflammatory cells (Fig. 5) and fewer Mikulicz cells (Fig. 5), there was lower pathogen burden (Sup. Fig 7)? In contrast to this data, the authors state, on page 13, that IL-10-/- mice were not capable of controlling the infection with dense highly inflammatory lesions. The authors go on to over interpret these findings stating that IL-10 is involved in the maturation of inflammatory monocytes, without any experimental data, and state that this points to direct evidence for the role of IL-10 in the acute->chronic transition, again without any evidence. These experiments should be re-interpreted, the language toned down and this section re-written. Similar to Figure 3, it would be useful to quantify the Mikulicz cells in the histology.
6. The data showing the bacterial load in the wild type and IL-10KO mice calls into question the functional relevance of the Mikulicz cells. Thus, the authors may wish to include it within the figures, rather than the supplement. Do the IL-10KO mice go on to clear the infection?
7. There were a few organizational and typographical errors in the manuscript:
a. Page 12: the data describing the splenectomized mice is shown in Supporting Information Fig 6, not Supporting Information Fig 5, as indicated in the text
b. Page 15: The Jackson Laboratory is located in Bar Harbor, Maine, USA
c. Page 18: Anne O'Garra's name is incorrectly written as Anne O'Gara
d. Page 25 and Page 10: The lower inoculum is listed as 2.106 in the text, but as 106 in the figure legend
e. Page 24: The letters described in the legend for Figure 1 do not correspond to the figure. For example, "A zoom into this region (G) revealed..." appears to be referring to F.
8. In the manuscript, there are a few minor points that are not clearly explained to the reader:
a. In several figures, symbols appeared within the axis titles.
b. As rhinoscleroma is not a commonly studied disease, it may benefit the reader to understand why there has previously been so little research into an animal model of rhinoscleroma and how this model differs to the previous mouse/ animal models mentioned.
c. Why are C57BL/6; BALB/c chimeric mice used as recipients in Figure 3, rather than a C57BL/6 → C57BL/6 transfer?
d. It is unclear throughout the manuscript which background of mouse (BALB/c or C57BL/6) is used for each experiment. This information should be added to the figure legends.
e. Page 7: it is not clear what an eccentric nucleus is
f. While it is stated in the Methods section that control mice are uninfected/saline-treated, this should be listed in the individual figures as well, for clarity

Referee #3 (Comments on Novelty/Model System):

Foamy macrophage formation is a common response to infections.

Referee #3 (General Remarks):

The author describe a murine model recapitulating the formation of Mikulicz cells following pulmonary K. rhinoscleromatis infection and show that Mikulicz cells are BM derived atypical inflammatory monocytes and depend in IL-10 of the host.

While the murine model is of interest, the following major issues should be considered:

1.Both BL6 and Balbc mice were used in this study, but there is no direct comparison of the two strains on the differential sensitivity to develop lung inflammation with Mikulicz cells upon K. rhinoscleromatis infection. The granuloma fromation is not described.
2.Foamy type of macrophages, now indentified as inflammatory macrophages type Mikulicz harboring bacteria occur commonly in lung infection including mycobacterial, not mentioned here. While IL-10 appears important for their development, other suppressive cytokines should be considered.
3.While the presence of IL-10 is required, it would be interesting to know whether supplementing BL6 mice or reconstitute IL-10 KO mice with exogenous IL-10 have an effect on Mikulicz cell recruitment and development.

4.While the presence of IL-10 is required, it would be interesting to know whether supplementing BL6 mice or reconstitute IL-10 KO mice with IL-10 has an effect on Mikulicz cell recruitment and development.
5.The functional properties of Mikulicz cells including neither the bactericidal property nor the biochemical properties of their vacuolar contents is analyzed.
6. The relevance of the mouse infection modeling human rhinosleroma has not been addressed, and the study is limited to the so-called Mikulicz cells, which id unlikely specific K. rhinoscleromatis infection.
7.The original source of the IL-10 deficient mice is not cited.

1st Revision - authors' response 31 January 2013

Referee #2

Question 1: 1. With the data presented, the authors do not definitively show that Mikulicz cells are exclusively inflammatory monocytes. For example, in Figure 2, the authors sort inflammatory monocytes and show that these cells have a Mikulicz cell phenotype, which does not rule out that cells expressing other (non-inflammatory monocyte) markers could have this phenotype as well. Have the authors sorted and done cytospins on the other cell populations described? Also, it is not clear from the data shown whether 100% of the inflammatory monocytes were Mikulicz cells or whether the Mikulicz cells were a subpopulation of total inflammatory monocytes. This is important, as the phrases "inflammatory monocytes" and "Mikulicz cells" are used interchangeably throughout the manuscript.

Response: We agree with the referee that our statement was not clearly demonstrated.

To substantiate our observation, we performed additional sorting of granulocytes, resident monocytes and inflammatory monocytes at three and five days post-infection with *K. rhinoscleromatis* and quantified the presence and the size of the Mikulicz cells in these three populations during infection. The results have been added to Figure 2 and are now described in the manuscript page 8. We thus observed that Mikulicz cells represent an atypical form of inflammatory monocytes that increase in size and number with time. Mikulicz cells were exclusively observed in the inflammatory monocyte population and never in the granulocyte and resident monocytes populations as summarized in the table below. We believe that this now shows that Mikulicz cells are a subpopulation of inflammatory monocytes that mature and increase in number over time during the infection up to representing almost all the inflammatory monocytes present in the lungs at day five, and that Mikulicz cells are exclusively a subpopulation of inflammatory monocytes.

In addition, we have restricted the use of inflammatory monocytes to the description of results based on FACS analysis, and Mikulicz cells to morphological microscopic observations.

	Percentage of Mikulicz cells in the subpopulation		
Days post-infection	Granulocytes	Resident monocytes	Inflammatory monocytes
3	0	0	44
5	0	0	98

Question 2. The FACS data shown in figure 3B would have more impact if the authors used the percentages to calculate the total numbers of inflammatory monocytes. For example, if the CCR2-/- mice have fewer total lung cell numbers, then there would be fewer total numbers of inflammatory monocytes. It is necessary to show total cell numbers to conclude that CCR2 is not involved in the

© EMBO

recruitment of these cells. Figure 3C could be improved by showing slides of both the wild type and CCR2-/- ; it would also be useful to quantify the numbers of Mikulicz cells by histology to confirm the phenotype shown by FACS and more conclusively exclude the involvement of CCR2 in their recruitment.

Response: We have analysed the data as suggested and included the total number of Mikulicz instead of percentage in Figure 3b and observed that there is no difference between the C56BL/6 and CCR2-/- mice. The figure has been modified to show representative image of histology (Figure 3C) as well as a quantification of the number of Mikulicz cells per mm2 of tissue in both WT and CCR2-/- mice (Figure 3D). Altogether, we believe that these new information clearly indicates that CCR2 is not involved in inflammatory monocytes recruitment during *K. rhinoscleromatis* infection nor their phenotypic maturation in Mikulicz cells.

Question 3. Can the authors add a representative histology image in Fig 3C of WT lungs, showing the presence of a similar frequency of Mikulicz cells.

Response: The figure has been modified to show representative image of histology of both WT and CCR2-/- mice in Figure 3C.

Question 4. There is only a subtle difference in the percentage of inflammatory monocytes shown in Figure 5. Fascinatingly, from the histological images, it appears that IL-10-/- mice have much fewer inflammatory cells. If total numbers of inflammatory monocytes were shown (by calculating from the total number of lung cells), would there be a greater difference between wild type and IL-10-/- mice?

Response: The data were analysed as suggested and we observed that whether inflammatory monocytes are expressed as percentage or total cell number per lungs does not change the interpretation of the data. We have specified in the manuscript that both percentage of inflammatory monocytes and total number of inflammatory monocytes are diminished in IL10 -/- mice and now present the data showing total number of inflammatory monocytes in Supplementary figure 7A.

Question 5: The interpretation for role of IL-10 in this model was very confusing. Despite there appearing to be fewer inflammatory cells (Fig. 5) and fewer Mikulicz cells (Fig. 5), there was lower pathogen burden (Sup. Fig 7)? In contrast to this data, the authors state, on page 13, that IL-10-/- mice were not capable of controlling the infection with dense highly inflammatory lesions. The authors go on to over interpret these findings stating that IL-10 is involved in the maturation of inflammatory monocytes, without any experimental data, and state that this points to direct evidence for the role of IL-10 in the acute->chronic transition, again without any evidence. These experiments should be re-interpreted, the language toned down and this section re-written. Similar to Figure 3, it would be useful to quantify the Mikulicz cells in the histology.

Response: We thank the referee for his comment on these experiments. We agree that our statement that “IL10-/- mice were not capable of controlling the infection” was misleading and that the overall description of this set of experiment could be improved. We have thus improved the description of the experiments with IL10-/- mice and mice injected with the IL10R antibody. As suggested, we have added a quantification of the number of Mikulicz cells on histology in IL10-/- mice and IL10R-treated mice (Figure 5C and Figure 6B). Both these experiments show that when IL10 is not produced or when the IL10 receptor is blocked by the antibody, 1) half the amount of inflammatory

© EMBO

monocyte is recruited to the lungs and 2) less than 10% of amount of Mikulicz cells are observed by histology. These experiments suggested that, because still 50% of inflammatory monocytes are present, IL10 participated directly or indirectly, in the phenotypic maturation of inflammatory monocytes, with a classical monocyte morphology, in typical Mikulicz cells.

We also acknowledge that our definition of Mikulicz cells maturation was not clear. We define Mikulicz cell maturation as the phenotypic morphologic change of classical inflammatory monocytes to the specific large highly vacuolated cells that Mikulicz cells typically are. This definition is now better demonstrated with the sorting experiment requested in question 1. This result and definition are now described page 8 in the results section showing Mikulicz cells are atypical inflammatory monocytes.

We also re-interpreted these IL10 results in the discussion and removed the mention to the role of IL10 in the acute-chronic transition. We hope the referee will now agree with our interpretation of these results.

Question 6. The data showing the bacterial load in the wild type and IL-10KO mice calls into question the functional relevance of the Mikulicz cells. Thus, the authors may wish to include it within the figures, rather than the supplement. Do the IL-10KO mice go on to clear the infection?

Response: To answer the referee question, as shown in Supporting Information Figure 7, IL10-/- mice are still heavily infected at day three post-infection. These mice are not able to clear the infection, and succumb from the infection between 3 and 4 days post-infection. On the contrary, WT mice survive up to 5 days post-infection with a high bacterial load (as shown in Figure 1).

We agree that the difference in bacterial load between WT and IL10-/- mice can suggest a functional role of these cells. However, we were not able to reach the same conclusion in mice injected with IL10R-blocking antibody. The bacterial load is not statistically significant between IgG control or IL10R antibody injected mice. The reason for such a difference is unknown but the two experimental conditions are different. Unlike the effects on inflammatory monocytes recruitment and Mikulicz cells maturation which are clearly observed in both experiments using IL10-/- mice or IL10R-injected mice, these experiments do not give the same conclusion on the control of the bacterial load, and, we believe, are thus not indicating a major role of IL10 in this process. Therefore, for clarity, we decided to keep the data showing the bacterial load in IL10-/- mice as Supporting Information Figure 7.

Moreover, we believe that the investigation of the functional properties of Mikulicz cells requires either to be able to specifically directly deplete them from the animal, while un-affecting the recruitment inflammatory monocytes, or to have an *in vitro* model in hand. Unfortunately, we are not aware of a mean of specifically depleting Mikulicz cells, and, despite our prolonged effort, we were not able to develop an *in vitro* model of Mikulicz cells. We have spent a lot of time and energy trying to develop such an in vitro model, by trying to have *K. rhinoscleromatis* phagocytosed by various murine and human monocytic and macrophage cell lines, whether they were stimulated or not with various cytokines including IL10, or conditioned with crude extracts of infected lungs. Despite all the conditions tested so far, we were unable to recapitulate Mikulicz cell formation *in vitro*. We believe that a deeper understanding of the animal model will help us find the conditions to develop such an *in vitro* model.

Question 7. There were a few organizational and typographical errors in the manuscript:

a. *Page 12: the data describing the splenectomized mice is shown in Supporting Information Fig 6, not Supporting Information Fig 5, as indicated in the text*

Response: This has been corrected. These data are now Supporting Information Figure 8.

b. Page 15: The Jackson Laboratory is located in Bar Harbor, Maine, USA

Response: The location of the Jackson Laboratory is now correctly spelled.

c. Page 18: Anne O'Garra's name is incorrectly written as Anne O'Gara

Response: Dr O'Garra's name is now correctly spelled in the acknowledgment.

d. Page 25 and Page 10: The lower inoculum is listed as 2.106 in the text, but as 106 in the figure legend

Response: The correct inoculum is 10^6. This has been corrected in the result section.

e. Page 24: The letters described in the legend for Figure 1 do not correspond to the figure. For example, "A zoom into this region (G) revealed..." appears to be referring to F.

Response: The figure legend has been corrected to mention the correct letter corresponding to the figures.

8. In the manuscript, there are a few minor points that are not clearly explained to the reader:

a. In several figures, symbols appeared within the axis titles.

Response: We have checked again all the figures for correct display of axis and symbols. They were all displayed properly in our file.

b. As rhinoscleroma is not a commonly studied disease, it may benefit the reader to understand why there has previously been so little research into an animal model of rhinoscleroma and how this model differs to the previous mouse/ animal models mentioned.

Response: There is no clear explanation to us as to why rhinoscleroma has attracted so little interest from the research community and we believe that this a very interesting model to study and better understand *Klebsiella* pathogenesis and the differential host response to the infection.

To develop a model allowing the study of rhinoscleroma pathogenesis, we wanted to use an animal model for which genetics was available and thus focused on mice. In this respect, even though we used an identical infection route as originally described by Steffen and Smith in 1961, our work completely re-established the murine model using different genetic backgrounds and significantly extended its characterisation. To help the reader understanding our work, we have added more

information in the beginning of the discussion of our model (page 12)

c. Why are C57BL/6; BALB/c chimeric mice used as recipients in Figure 3, rather than a C57BL/6 → C57BL/6 transfer?

Response: We used these chimeras because we wanted to have a partial BALB/c genetic background for this experiment. Indeed BALB/c mice present a higher number of inflammatory monocytes and Mikulicz cells than C57BL/6 mice (data now shown in Supporting Information Figure 2); they provide qualitatively better observations and carry the CD45.2 marker. As the donor mice carrying the CD45.1 marker we had were on a C57BL/6 background, we therefore decided to use BALB/c;C57BL/6 chimera. This information has been added to the description of the results.

d. It is unclear throughout the manuscript which background of mouse (BALB/c or C57BL/6) is used for each experiment. This information should be added to the figure legends.

Response: The background of the mice used has been added to the figure legends and within the text.

e. Page 7: it is not clear what an eccentric nucleus is.

Response: This qualification has been replaced by "a nucleus generally visible on the side of the cell" in page 7.

f. While it is stated in the Methods section that control mice are uninfected/saline-treated, this should be listed in the individual figures as well, for clarity

Response: When needed, "saline-injected controls" has been added to the figure legends.

Referee #3 (Comments on Novelty/Model System):

Foamy macrophage formation is a common response to infections.

Response: We agree with the referee that foamy macrophages are common features of several infectious diseases and that this term is misleading when referring to Mikulicz cells. Generally, one refers to foamy macrophages encountered in metabolic diseases (atherosclerosis) or infectious diseases with pathogens like *Mycobacterium tuberculosis*, *M leprae*, *Chlamydia pneumoniae* or *Toxoplasma gondii*. In all these infectious diseases, foamy macrophages have bacteria-containing phagosomes together with lipid vesicles. These vesicles are what give the foamy aspect to these macrophages. They arise as the results of a dysregulation of transport of lipids from low-density lipoproteins particles. By electron microscopy, these lipids are easily seen as electron dense

intracellular vesicles (for a review see for example (Russell *et al*, 2009).

However, Mikulicz cells, as they are described in the literature, are different from this type of foamy macrophages. They are specific to rhinoscleroma and have distinctive features to other foamy macrophages as in tuberculosis for instance. They have larger vacuoles that look extremely 'bright' on histological section, like empty vacuoles. By electron microscopy, they do not present this intense electron staining as observed with lipid droplets and are bacteria-containing phagocytic vacuoles. In addition, our unpublished observation rather indicates that these vacuoles are enriched in carbohydrate molecules as suggested by Hoffmann (Hoffmann *et al*, 1973). For all, these reasons, and to the best of our knowledge, Mikulicz cells encountered in rhinoscleroma are different from other foamy macrophages observed in other infectious diseases.

We have discussed the difference between foamy macrophages and Mikulicz cells at the beginning of the discussion.

Referee #3 (General Remarks):

The author describe a murine model recapitulating the formation of Mikulicz cells following pulmonary K. rhinoscleromatis infection and show that Mikulicz cells are BM derived atypical inflammatory monocytes and depend in IL-10 of the host.

While the murine model is of interest, the following major issues should be considered:

1.Both BL6 and Balbc mice were used in this study, but there is no direct comparison of the two strains on the differential sensitivity to develop lung inflammation with Mikulicz cells upon K. rhinoscleromatis infection. The granuloma formation is not described.

Response: We have added data showing the comparison of BALB/c vs C57BL/6 mice in their response to *K. rhinoscleromatis* infection (Supporting Information Figure 2).

As stated in the text, our model recapitulates an "acute" state of the disease, and in this conditions, we were not able to observe any granuloma formation. It is yet not clear whether the mouse model is adapted to the study of granuloma formation in this disease. Steffen and Smith (1961) and us (our unpublished observation) were not able to detect any granuloma in mice. But granuloma has been reported in rat and rabbit (Gaafar *et al*, 2000; Talaat *et al*, 1978). This is stated in the discussion p12. However, we agree that this is an important aspect of the disease that deserves to be investigated in separated studies using a different animal model.

2.Foamy type of macrophages, now identified as inflammatory macrophages type Mikulicz harbouring bacteria occur commonly in lung infection including mycobacterial, not mentioned here. While IL-10 appears important for their development, other suppressive cytokines should be considered.

Response: We agree with the referee comment that other suppressive cytokines are likely to be involved in the development of Mikulicz cells. However, we believe that our data clearly show a major role of IL10 in this process as almost no Mikulicz cells are detected in IL10-/- mice and thus point to an essential function of this cytokine in this process. The role of such other cytokine could be specifically analysed in an additional study.

3.While the presence of IL-10 is required, it would be interesting to know whether supplementing

BL6 mice or reconstitute IL-10 KO mice with exogenous IL-10 have an effect on Mikulicz cell recruitment and development.

4.While the presence of IL-10 is required, it would be interesting to know whether supplementing BL6 mice or reconstitute IL-10 KO mice with IL-10 has an effect on Mikulicz cell recruitment and development.

Response: We agree that these experiments could be interesting. However, we believe that we have provided strong enough evidences on the role of IL10 in the recruitment and maturation of Mikulicz cells by using two different experimental approaches. Moreover, the IL10-deficient mice we used have already been utilized in several infectious studies (Loebbermann *et al*, 2012; Grünig *et al*, 1997; Bai *et al*, 2009; Couper *et al*, 2008; Gazzinelli *et al*, 1996; Redford *et al*, 2010). For all of these experiments, the proper control was the use of WT mice, and no complementation studies were performed. While this kind of experiment is common in *in vitro* studies such as complementing bacteria mutants, it is, to our knowledge, uncommon with knockout mice. In addition, we used as control mice, BALB/c mice that were specifically provided to us by Anne O'Garra. These mice were used to generate this IL10 KO strain in this genetic background and were bred in a similar manner as the IL10-/- mice. We are therefore confident that the best control possible for these specific experiments was used and believe that complementing IL10 KO mice will not substantially add to the results presented in this manuscript.

5.The functional properties of Mikulicz cells including neither the bactericidal property nor the biochemical properties of their vacuolar contents is analysed.

Response: We agree with the referee that investigating the functional properties of Mikulicz cells is an important question. As indicated above to the question 5 from referee 2, due to the current constraints of our animal model (we can only isolate fixed pure Mikulicz cells because of their extreme fragility), we cannot address specifically these questions. We paste below the answer provided as it also pertains to this comment.

We believe that the investigation of the functional properties of Mikulicz cells requires either to be able to specifically directly deplete them from the animal, while un-affecting the recruitment inflammatory monocytes, or to have an *in vitro* model in hand. Unfortunately, we are not aware of a mean of specifically depleting Mikulicz cells, and, despite our prolonged effort, we were not able to develop an *in vitro* model of Mikulicz cells. We have spent a lot of time and energy trying to develop such an in vitro model, by trying to have *K. rhinoscleromatis* phagocytosed by various murine and human monocytic and macrophage cell lines, whether they were stimulated or not with various cytokines including IL10, or conditioned with crude extracts of infected lungs. Despite all the conditions tested so far, we were unable to recapitulate Mikulicz cell formation *in vitro*. We believe that a deeper understanding of the animal model will help us find the conditions to develop such an *in vitro* model. . It is our current objective to develop such an *in vitro* model and we hope to be able to answer these questions in future study.

6. The relevance of the mouse infection modelling human rhinosleroma has not been addressed, and the study is limited to the so-called Mikulicz cells, which id unlikely specific K. rhinoscleromatis infection.

Response: As stated above, to the best of our knowledge, Mikulicz cells are different from other foamy macrophages and are specific to rhinoscleroma. As indicated in the discussion, the mouse model does recapitulate a characteristic feature of the human disease, the occurrence of Mikulicz cells, which is the focus of our present work.

© EMBO

7.The original source of the IL-10 deficient mice is not cited.

Response: The mice were obtained from Anne O'Garra with permission of Werner Muller who generated these mice. This is indicated in the material and methods section.

References

Bai F, Town T, Qian F, Wang P, Kamanaka M, Connolly TM, Gate D, Montgomery RR, Flavell RA & Fikrig E (2009) IL-10 signaling blockade controls murine West Nile virus infection. *PLoS Pathog* 5: e1000610

Couper KN, Blount DG, Wilson MS, Hafalla JC, Belkaid Y, Kamanaka M, Flavell RA, de Souza JB & Riley EM (2008) IL-10 from CD4CD25Foxp3CD127 adaptive regulatory T cells modulates parasite clearance and pathology during malaria infection. *PLoS Pathog* 4: e1000004

Gaafar HA, Bassiouny M, Mofty El M, Badour NM & Nour YA (2000) Experimental intravenous inoculation of Klebsiella rhinoscleromatis bacilli in albino rats: a histopathological and bacteriological study. *Acta Otolaryngol* 120: 279–285

Gazzinelli RT, Wysocka M, Hieny S, Scharton-Kersten T, Cheever A, Kühn R, Muller W, Trinchieri G & Sher A (1996) In the absence of endogenous IL-10, mice acutely infected with Toxoplasma gondii succumb to a lethal immune response dependent on CD4+ T cells and accompanied by overproduction of IL-12, IFN-gamma and TNF-alpha. *J Immunol* 157: 798–805

Grünig G, Corry DB, Leach MW, Seymour BW, Kurup VP & Rennick DM (1997) Interleukin-10 is a natural suppressor of cytokine production and inflammation in a murine model of allergic bronchopulmonary aspergillosis. *J Exp Med* 185: 1089–1099

Hoffmann EO, Loose LD & Harkin JC (1973) The Mikulicz cell in rhinoscleroma. Light, fluorescent and electron microscopic studies. *Am J Pathol* 73: 47–58

Loebbermann J, Schnoeller C, Thornton H, Durant L, Sweeney NP, Schuijs M, O'Garra A, Johansson C & Openshaw PJ (2012) IL-10 regulates viral lung immunopathology during acute respiratory syncytial virus infection in mice. *PLoS ONE* 7: e32371

Redford PS, Boonstra A, Read S, Pitt J, Graham C, Stavropoulos E, Bancroft GJ & O'Garra A (2010) Enhanced protection to Mycobacterium tuberculosis infection in IL-10-deficient mice is accompanied by early and enhanced Th1 responses in the lung. *Eur J Immunol* 40: 2200–2210

Russell DG, Cardona P-J, Kim M-J, Allain S & Altare F (2009) Foamy macrophages and the progression of the human tuberculosis granuloma. *Nat Immunol* 10: 943–948

Steffen TN & Smith IM (1961) Scleroma. Klebsiella rhinoscleromatis and its effect on mice. *Ann Otol Rhinol Laryngol* 70: 935–952

Talaat M, Soliman A, Gaafar H & Belal A (1978) Experimental scleroma. A histopathological study. *J Laryngol Otol* 92: 489–498

Acceptance 14 February 2013

Please find enclosed the final reports on your manuscript. We are pleased to inform you that your manuscript is accepted for publication and is being sent to our publisher to be included in the next available issue of EMBO Molecular Medicine.

Congratulations on your interesting work.

© EMBO

***** Reviewer's comments *****

Referee #3 (Comments on Novelty/Model System):

The described model recapitulates some aspects of the clinical rhinosclerome, however the nature of Mickulicz cells is not fully understood.

Referee #3 (General Remarks):

It is a solid contribution, which has however its limitation.
The authors replied to the concerns raised by the referees and improved the quality of paper.
