# Supplementary material for: A novel murine model of rhinoscleroma identifies Mikulicz cells, the disease signature, as IL-10 dependent derivatives of inflammatory monocytes
Source: EMBO Mol Med. 2013 Apr 2;5(4):516–30. doi: 10.1002/emmm.201202023 (PMC3628109; doi:10.1002/emmm.201202023)
Supplement: Supplementary file 2 [file emmm0005-0516-sd2.pdf]

Table of Contents

Supporting Information Figure 1	2
Supporting Information Figure 2	3
Supporting Information Figure 3	4
Supporting Information Figure 4	5
Supporting Information Figure 5	6
Supporting Information Figure 6	8
Supporting Information Figure 7	9
Supporting Information Figure 8	10

Supporting Information Figure 1

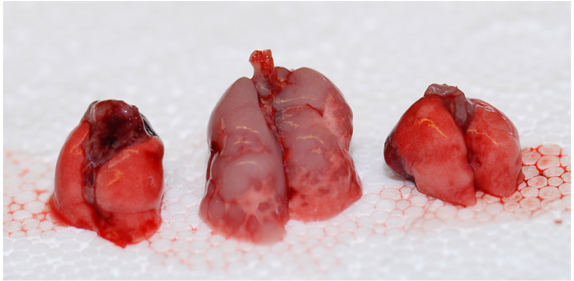

Macroscopic aspect of lungs of BALB/c mice that have been saline-injected (left), or infected with 2.10^7 *K. rhinoscleromatis* (middle) or 2.10^4 Kp52.145 (right) five days post-infection.

Supporting Information Figure 2

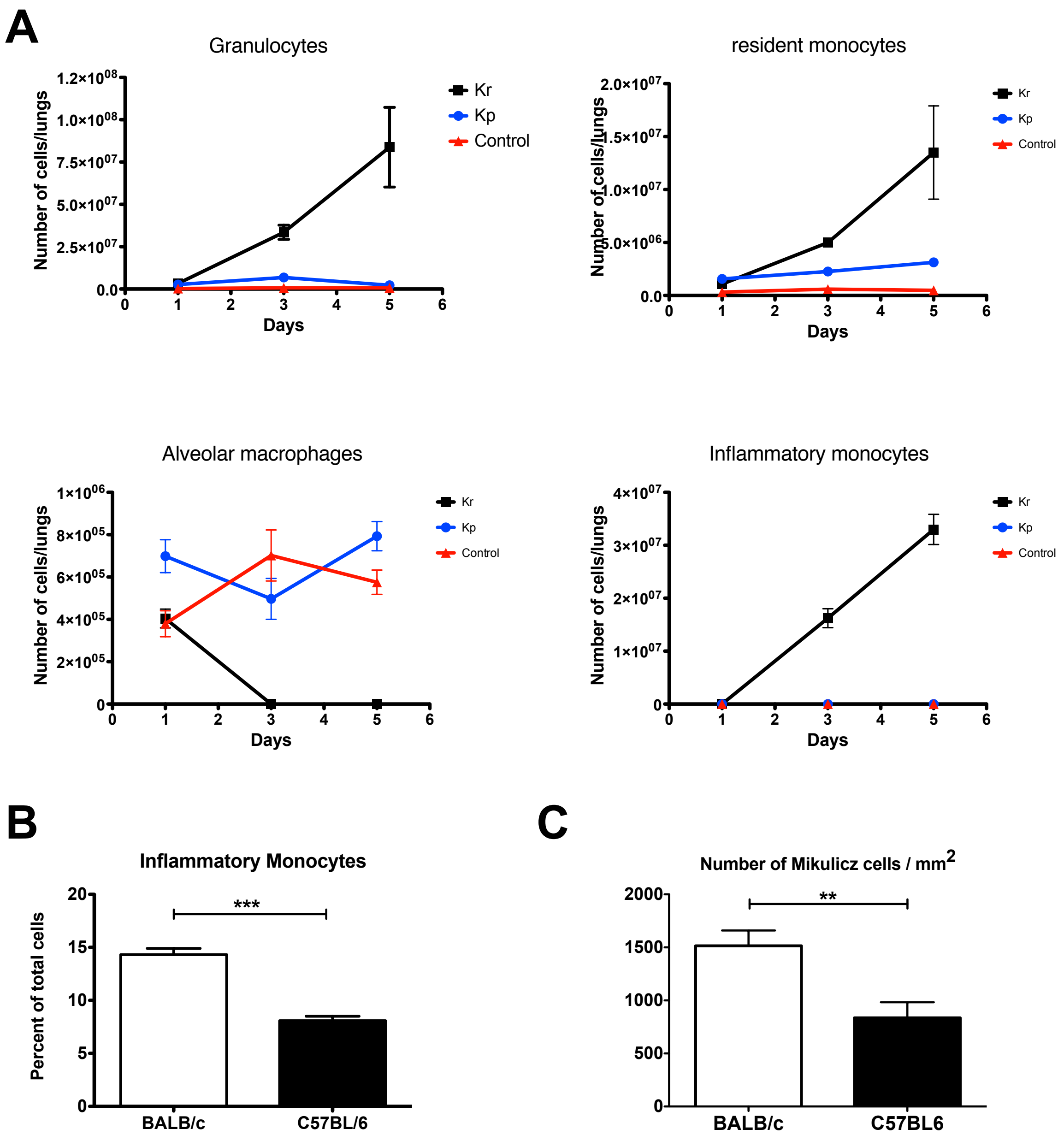

A) Kinetic of cells recruitment after infection with *K. rhinoscleromatis* and Kp52.145. Lung cells of BALB/c mice infected with 2.10^7 *K. rhinoscleromatis*, 2.10^4 Kp52.145 or saline-injected controls were isolated 1, 3 and 5 days post inoculation and stained for granulocytes (Gr1+ F4/80- CD11b+ CD11c-), resident monocytes (Gr1- F4/80+ CD11b+ CD11c-), alveolar macrophages (Gr1- F4/80+ CD11b- CD11c+) or inflammatory monocytes (Gr1+ F4/80+ CD11b+ CD11c-). Results show the number of each cell population in the total lung cells. Data are mean +/- sem and represent between 6 and 12 mice for each point from at least three independent experiments.

B) Comparison of percentage of inflammatory monocytes between BALB/c and C57BL/6 mice infected with 2.10^7 *K. rhinoscleromatis* at three days post-infection. Data are mean +/- sem. (*** , p<0,0001)

C) Comparison of number of Mikulicz cells in tissue sections between BALB/c and C57BL/6 mice infected with 2.10^7 *K. rhinoscleromatis*. Data are mean +/- sem and represent between 10 and 16 measurements. (**, p=0,0045)

Supporting Information Figure 3

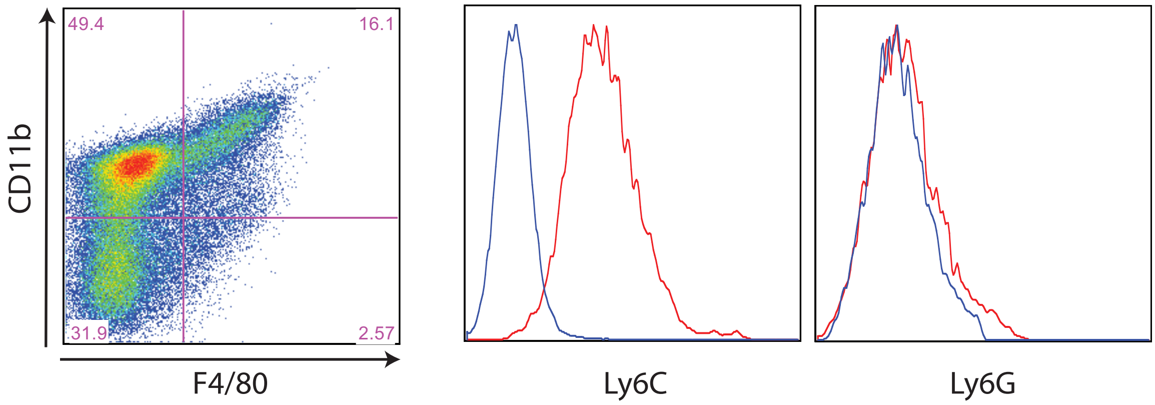

Characterisation of inflammatory monocytes by FACS: Inflammatory monocytes isolated from lungs of *K. rhinoscleromatis* infected BALB/c mice were labelled with CD11b and F4/80 antibodies. Double positive cells (upper right quadrant) were analysed for their expression of Ly6C and Ly6G (red) as compared to isotype control (blue). Inflammatory monocytes are Ly6C positive and Ly6G negative.

Supporting Information Figure 4

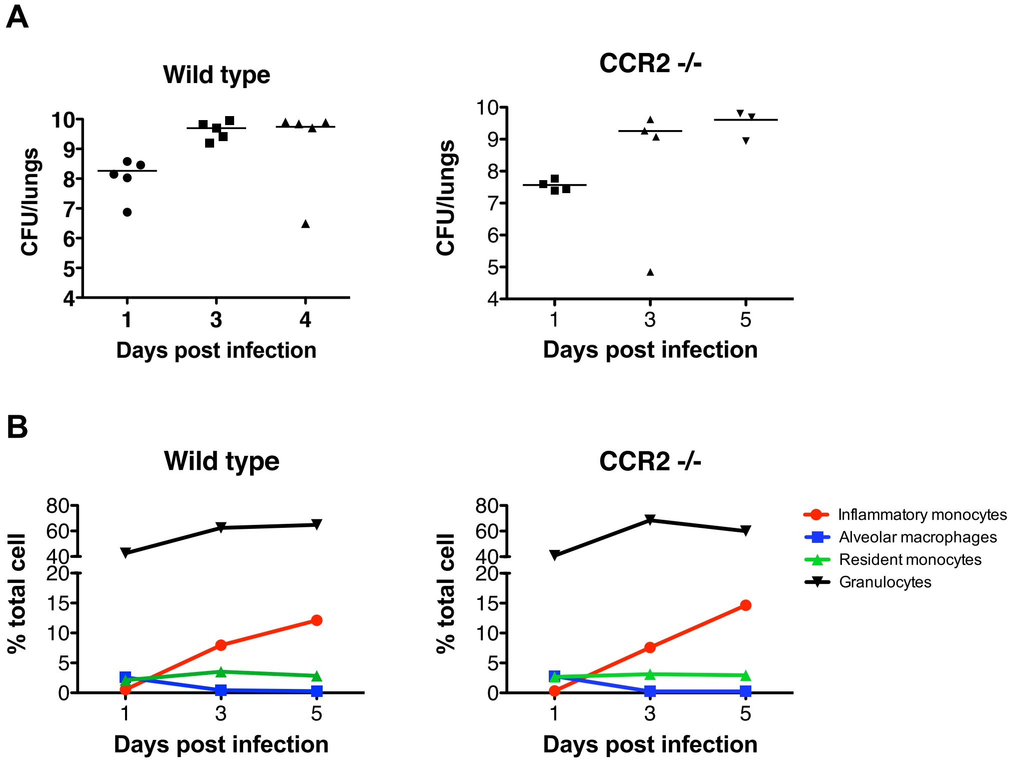

Infection of CCR2-/- mice

A) Bacterial load in lungs of C57BL/6 CCR2-/- (right) or C57BL/6 WT mice (left) infected with 2.10^7 *K. rhinoscleromatis*. Data show log CFU/organ.

B) Kinetics of cell recruitment after infection with *K. rhinoscleromatis* of C57BL/6 CCR2-/- (right) or C57BL/6 WT mice (left). Data are mean +/-sem from 3 to 14 mice from 2 independent experiments.

Supporting Information Figure 5

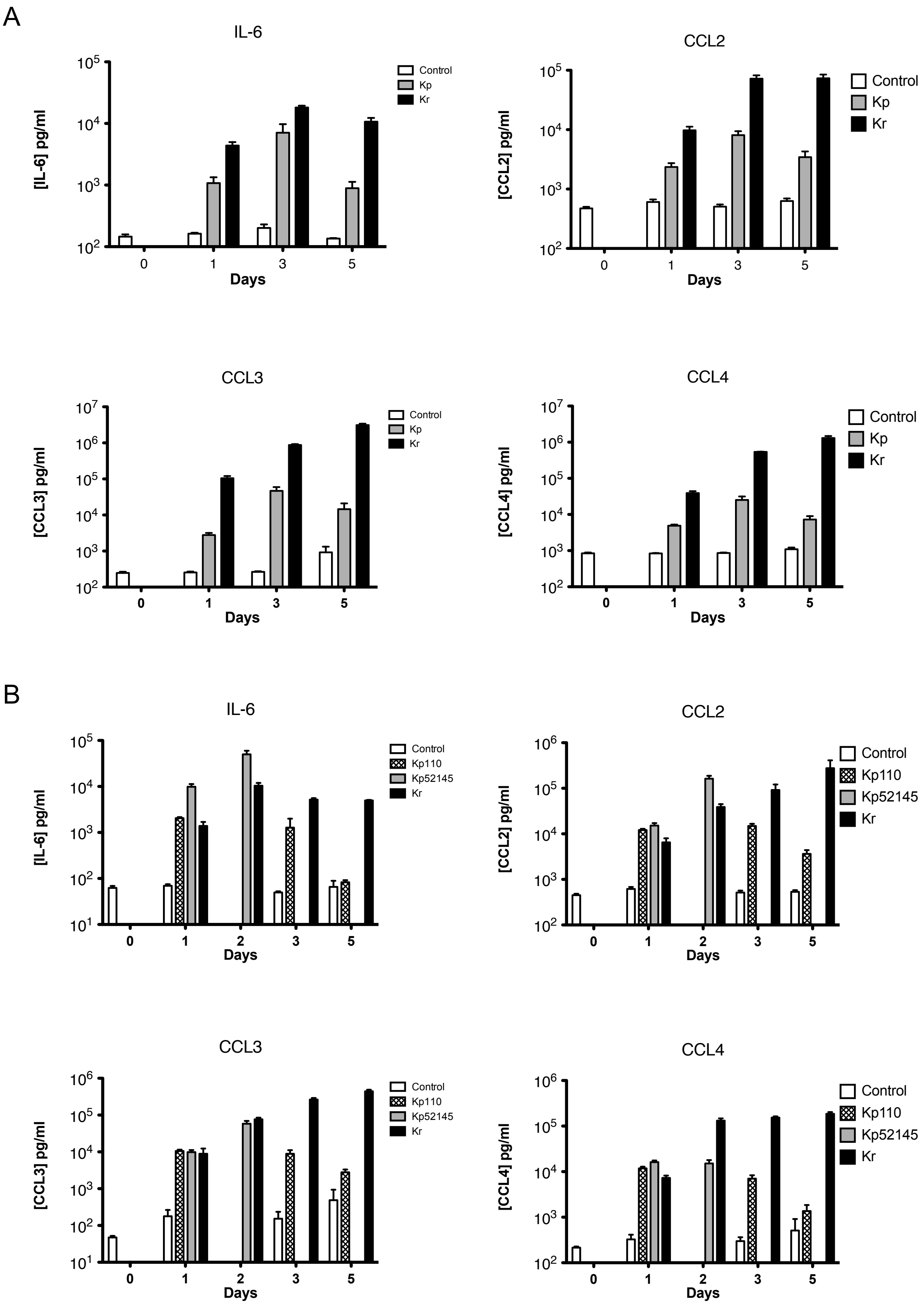

Production of IL-6, CCL2, CCL3, CCL4, in the lung of BALB/c mice infected by *K. rhinoscleromatis*, Kp52.145 or Kp110.

(A) Mice were infected with 2.10^7 *K. rhinoscleromatis* or 2.10^4 Kp52.145 or saline-injected. One, three and five days post-infection their lungs were homogenized and the cytokines and chemokines were measured in the extracts by ELISA. Data are mean +/- sem from 8 to 15 mice from 2 independent experiments.
(B) Mice were infected with 2.10^7 *K. rhinoscleromatis*, Kp52.145 or Kp110 strains. Cytokines were measured at different days post infection (1, 2, 3 and 5 for *K. rhinoscleromatis*; 1, 3 and 5 for Kp110; 1 and 2 for Kp52.145). Data are mean +/- sem from 3 to 9 mice from 2 independent experiments.

Supporting Information Figure 6

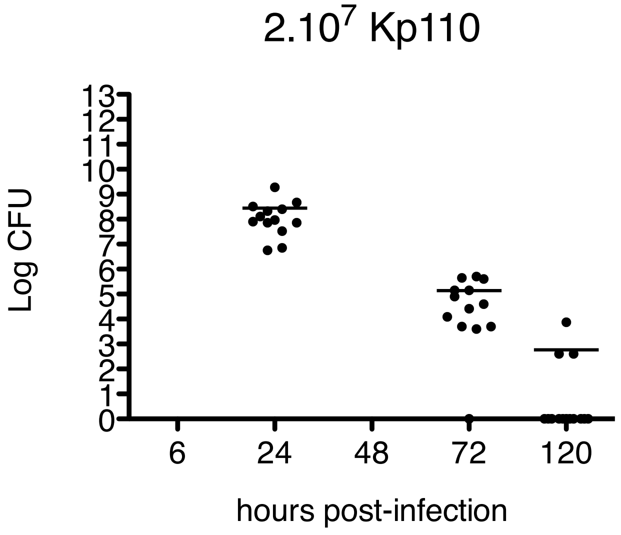

Bacterial load in lungs of BALB/c mice after infection with 2.10^7 Kp110. CFU in whole lungs after 1, 2, 3 or 5 days post-infection.

Supporting Information Figure 7

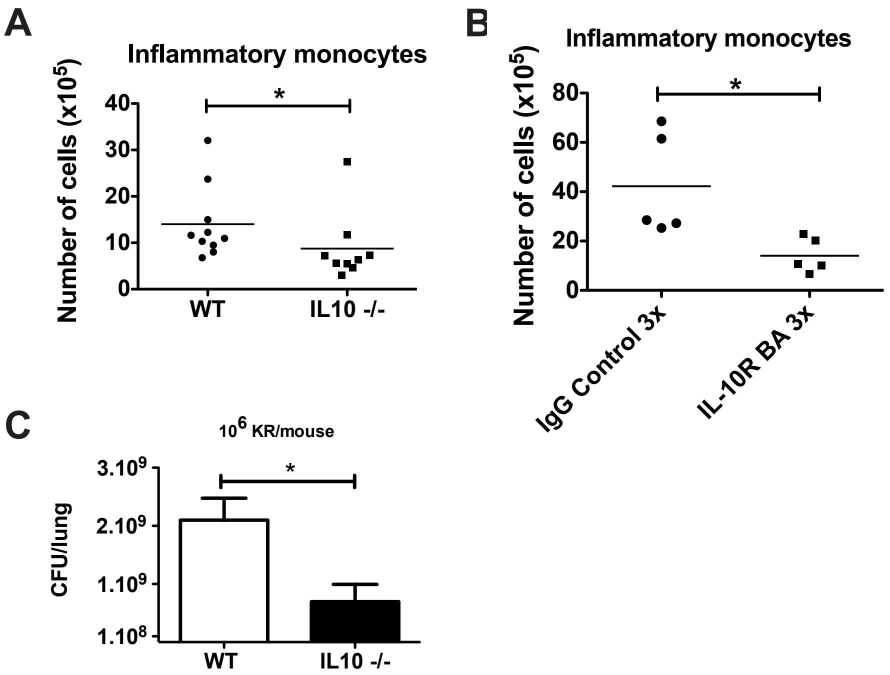

A) Number of inflammatory monocytes in lungs of BALB/c WT and IL10-/- mice three days post-infection with 10^6 *K. rhinoscleromatis*. (*, p=0,02).
B) Number of inflammatory monocytes in lungs of BALB/c mice injected at day 1, 2 and 3 post-infection with 100 μg of control igG or anti-IL10R antibody. CFU are determined four days post-infection with 10^6 *K. rhinoscleromatis*. (*, p=0,022).
C) CFU per lungs in WT and IL-10-/- mice.
Mice were infected with 10^6 *K. rhinoscleromatis* and CFU were numerated 3 days post-infection. In IL-10-/- mice the recovered bacterial load was about one third of what is observed in WT mice (6.9 10^8 versus 2.1 10^9 bacteria/lung). Data show mean+/- sem from 7 to 8 mice from 2 independent experiments.(*, p=0,013)

Supporting Information Figure 8

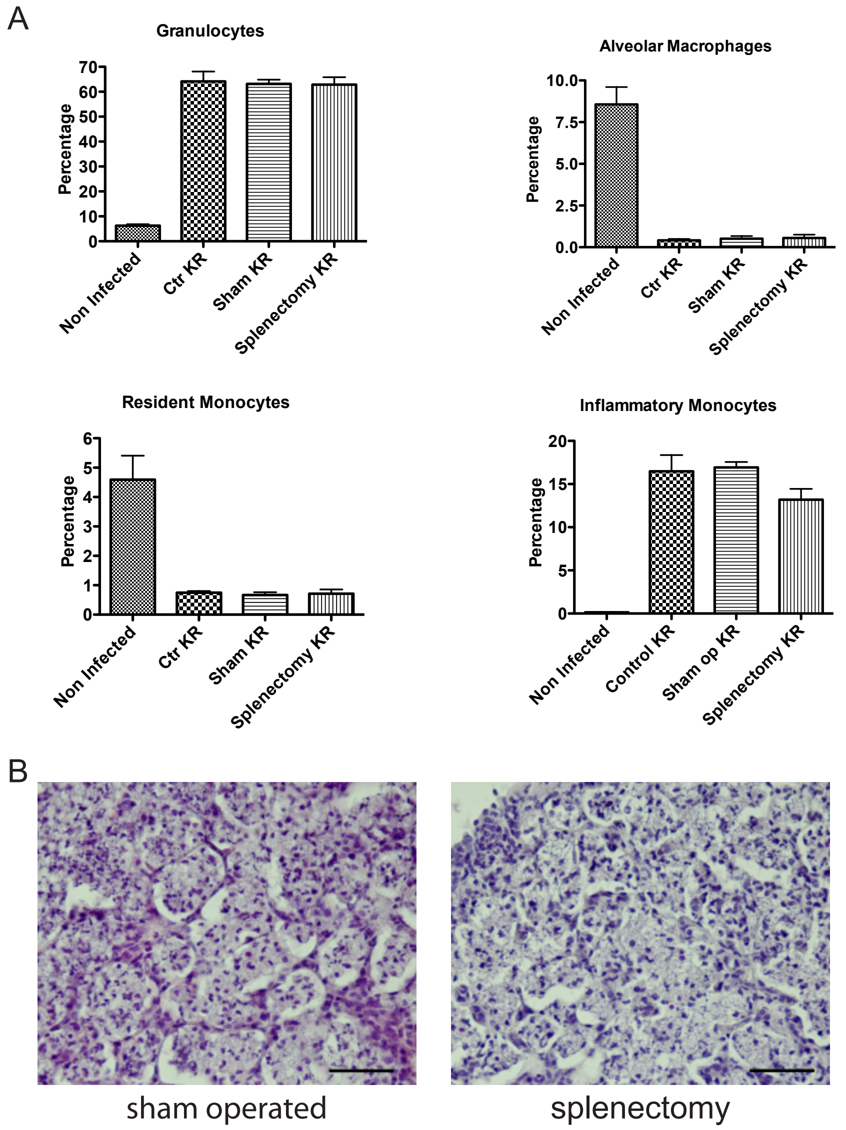

Splenic monocytes are not the main source of Mikulicz cells.

A) Percentage of granulocytes, alveolar macrophages, resident monocytes and inflammatory monocytes in lungs of control mice, sham-operated or splenectomised mice 4 days post-infection. The percentage of inflammatory monocytes in splenectomised mice is reduced by 20% as compared to sham-operated mice. Data are mean+/- sem from 4 mice from 2 independent experiments.

B) Presence of Mikulicz cells in sham operated mice and splenectomised mice. Scale bar is 100 μm.
